# BCMA-targeted immunotherapy for multiple myeloma

**DOI:** 10.1186/s13045-020-00962-7

**Published:** 2020-09-17

**Authors:** Bo Yu, Tianbo Jiang, Delong Liu

**Affiliations:** 1 Department of Medicine, Lincoln Medical Center, Bronx, NY USA; 2 Department of Medicine, New York Medical College and Westchester Medical Center, Valhalla, NY USA

**Keywords:** B cell maturation antigen, BCMA, Belantamab mafodotin, CAR-T, Antibody-drug conjugate, Bispecific T cell engager

## Abstract

B cell maturation antigen (BCMA) is a novel treatment target for multiple myeloma (MM) due to its highly selective expression in malignant plasma cells (PCs). Multiple BCMA-targeted therapeutics, including antibody-drug conjugates (ADC), chimeric antigen receptor (CAR)-T cells, and bispecific T cell engagers (BiTE), have achieved remarkable clinical response in patients with relapsed and refractory MM. Belantamab mafodotin-blmf (GSK2857916), a BCMA-targeted ADC, has just been approved for highly refractory MM. In this article, we summarized the molecular and physiological properties of BCMA as well as BCMA-targeted immunotherapeutic agents in different stages of clinical development.

## Introduction

Recent advances in novel therapeutics such as proteasome inhibitors (PI) and immunomodulatory drugs (IMiD) have significantly improved the treatment outcomes in patients with multiple myeloma (MM) [1–8]. However, most MM patients eventually relapse due to the development of drug resistance [9]. In addition, many of the current popular target antigens, such as CD38 and SLAMF7 (also known as CS1 or CD319), are also found in other normal tissues, thus leading to unwanted off-tumor toxicities [10, 11]. Therefore, novel treatment strategies are urgently needed, especially in high-risk relapsed/refractory (R/R) MM [12–15]. B cell maturation antigen (BCMA) or CD269, also known as tumor necrosis factor receptor superfamily member 17 (TNFRSF-17), is restrictively expressed in both normal and malignant plasma cells (PC) at high levels, which makes it an ideal target antigen for novel MM therapies [16, 17].

## B cell maturation antigen (BCMA)

**Fig. 1:**
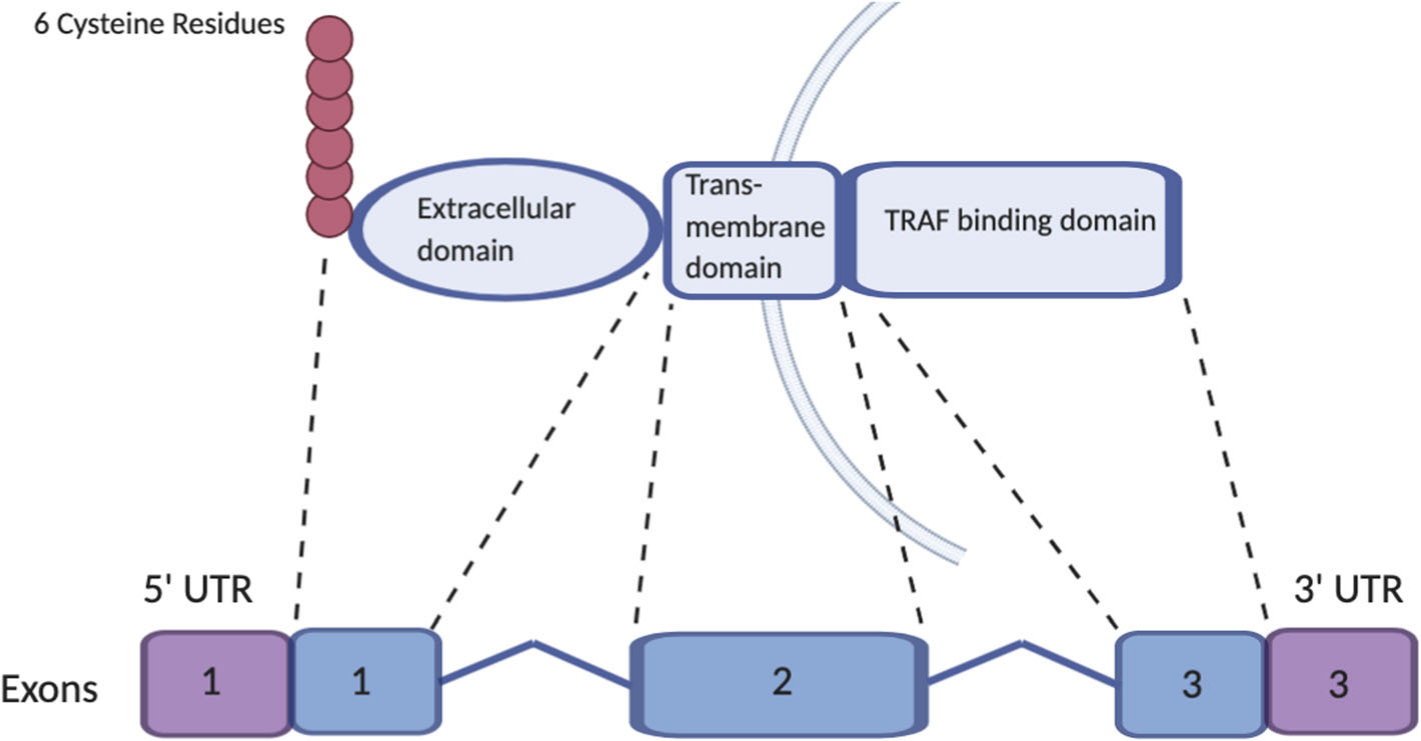
BCMA gene and protein. BCMA is encoded by the *TNFRSF17* gene (BCMA gene) located on the short arm of chromosome 16 (16p13.13). The BCMA gene comprised of 3 exons separated by 2 introns. BCMA is a type III transmembrane glycoprotein, with an extracellular N terminus containing a conserved motif of 6 cysteines and an intracellular tumor necrosis factor receptor-associated factor (TRAF) binding domain that triggers the activation of nuclear factor κ-light-chain enhancer of activated B cells (NF-κβ) signaling

BCMA is encoded by a 2.92-kb *TNFRSF17* gene located on the short arm of chromosome 16 (16p13.13) and composed of 3 exons separated by 2 introns (Fig. 1). BCMA is a 184 amino acid and 20.2-kDa type III transmembrane glycoprotein, with the extracellular N terminus containing a conserved motif of 6 cysteines [18–21]. BCMA was found to be a member of tumor necrosis factor (TNF) receptor (TNFR) superfamily [22]. There are four natural splice variants of human BCMA that present with different receptor binding affinities, membrane-anchoring ability, and intracellular domain signaling [19, 23].

BCMA, along with the other two functionally related TNFR superfamily members, B cell activating factor (BAFF; also called BLyS) receptor (BAFF-R) and transmembrane activator and calcium modulator and cyclophilin ligand interactor (TACI), coordinates to regulate B cell proliferation maturation and survival, as well as differentiation into plasma cells (PCs) [24–30]. Unlike BAFF-R and TACI, BCMA is almost exclusively expressed on plasmablasts [31] and PCs [32]. It is also weakly detectable on some memory B cells committed to PC differentiation and on plasmacytoid dendritic cells [33]. BCMA is undetectable in naïve B cells, hematopoietic stem cells, or in normal non-hematologic tissues except for some organs such as the testis, trachea, and some portions of gastrointestinal duct due to the presence of PCs [34]. The upregulation of BCMA is induced by B lymphocyte-induced maturation protein1 (Blimp-1), an essential transcription factor involved in the development and survival of PCs [35]. In BCMA−/− mice, the long-term survival of PCs is impaired, but lack of BCMA has no effect in short-lived PCs, B cell development, or early humoral immune response, and the splenic architecture and germinal centers appear intact in these BCMA-deficient mice [32, 36]. Therefore, the presence of BCMA might be specifically required to enhance the survival of long-lived PCs.

Meanwhile, BCMA is identified on the surface of nearly all MM cell lines (80–100%) and is more abundantly present in malignant PCs than normal PCs [16, 37]. MM patients receiving allogeneic transplant often develop donor-derived anti-BCMA monoclonal antibodies (mAbs) after donor lymphocyte infusion and benefit from graft-versus-tumor response [38]. In contrast, TACI is expressed at a significantly lower concentration and BAFF-R is even hardly detectable on MM cells [39]. BCMA overexpression significantly promotes in vivo growth of xenografted MM cells in murine models [40]. Furthermore, BCMA expression is upregulated during MM pathogenesis and evolution, from normal to MGUS to SMM to active MM [41]. Higher levels of BCMA are associated with poorer outcomes [16], indicating that BCMA is a useful biomarker of disease activity and prognosis for MM.

**Fig. 2.**
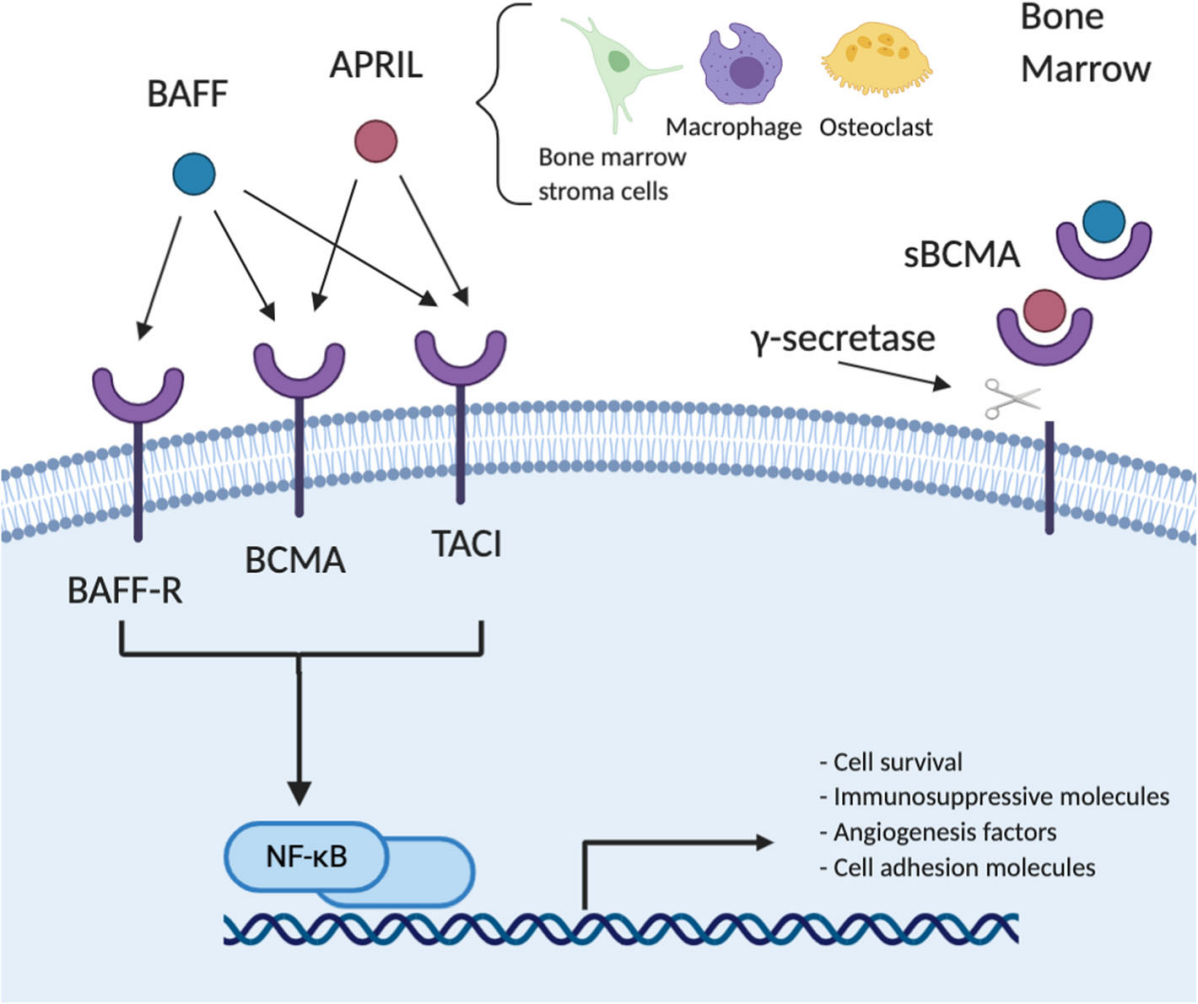
BCMA signaling pathway. BCMA has two agonist ligands: a proliferation-inducing ligand (APRIL) and B cell activating factor (BAFF), which are mainly secreted by the bone marrow (BM) stromal cells, osteoclasts, and macrophages in a paracrine manner in the BM. APRIL exhibits a much higher binding affinity to BCMA than BAFF, and it also binds to TACI, while BAFF endorses more selectivity to BAFF-R. Multiple growth and survival signaling cascades are subsequently activated in the multiple myeloma (MM) cells, most frequently through NF-κβ, leading to upregulation of anti-apoptotic proteins and production of cell adhesion molecules, angiogenesis factors, and immunosuppressive molecules. These lead to increased survival of MM cells. Membrane BCMA can be cleaved by γ-secretase and released to the plasma as soluble BCMA (sBCMA). sBCMA can bind to APRIL and BAFF, which may interfere with the activation of BCMA signaling pathways

BCMA has two agonist ligands: a proliferation-inducing ligand (APRIL) and BAFF, which are mainly secreted by bone marrow (BM) stromal cells, osteoclasts, and macrophages in a paracrine manner in the BM [40, 42–44] (Fig. 2). APRIL exhibits a much higher binding affinity to BCMA than BAFF [45], and it also binds to TACI [46], while BAFF restricts more selectivity to BAFF-R [45]. Thus, APRIL is more specific to PCs and correlates to more downstream pathophysiological activities [47]. MM cell lines had significantly reduced growth when xenografted in APRIL−/− mice [48]. In MM patients, the serum levels of APRIL and BAFF are elevated about 5-fold over those in the healthy controls [42], and the more advanced the stage of MM is, the higher concentration of ligands is detected [49]. Studies have shown that MM cells could stimulate osteoclasts to produce more APRIL which contributes to an immunosuppressive BM microenvironment [40, 50]. It has been suggested that adding APRIL blocking monoclonal antibodies (mAbs) to BCMA-directed immunotherapies might overcome MM cell-induced immunosuppressive BM microenvironment and thereby enhance the antibody-dependent cell-mediated cytotoxicity (ADCC) against MM cells [51].

Upon binding of the ligands to BCMA, multiple growth and survival signaling cascades are activated in MM cells, most frequently nuclear factor κ-light-chain enhancer of activated B cells (NF-κβ), but also including rat sarcoma/mitogen-activated protein kinase (RAS/MAPK), and phosphoinositide-3-kinase–protein kinase B/Akt (PI3K-PKB/Akt) signaling pathway [37, 52, 53]. These pathways result in proliferation stimulation by modulating cell cycle checkpoints, increased survival by upregulating anti-apoptotic proteins (e.g., Mcl-1, BCL-2, BCL-XL), and production of cell adhesion molecules (e.g., ICAM-I), angiogenesis factors (e.g., VEGF, IL-8), and immunosuppressive molecules (e.g., IL-10, PD-L1, TGF-β) [37, 40, 54]. In vitro studies have shown that BCMA overexpression can even trigger the activation of NF-κβ and MAPK pathways in MM cells itself without stimulation of APRIL or BAFF [55]. In addition, there are many cross-talks between APRIL/BCMA signaling and other pathways. For example, APRIL interacts with CD138/syndecan-1 and heparan sulfate proteoglycans (HSPG) to promote proliferation and survival of MM cells [56]. Concomitant blockade of FGF-R3 and JAK2 leads to BCMA downregulation [57]. In vitro study also shows that BCMA co-immunoprecipitates with interferon regulatory factor-4 (IRF-4), a master transcription factor mediating survival of MM cells, further emphasizing its role in the oncogenesis of MM [58].

## Soluble BCMA (sBCMA)

BCMA has a soluble form, sBCMA, derived from direct shedding of the membrane BCMA through γ-secretase activity. sBCMA retains the extracellular domain and a part of the transmembrane region [59]. sBCMA represents a potential biomarker for B cell involvement in human autoimmune diseases such as systemic lupus erythematosus, rheumatoid arthritis, and multiple sclerosis [59, 60]. In MM patients, the serum level of sBCMA is found to be significantly elevated compared to healthy individuals [61]. A higher serum level of sBCMA independently correlates to a heavier disease burden, a worse clinical and radiological response, and a poorer prognosis [62]. A remarkable decrease in sBCMA level was observed in patients with good responses to BCMA-targeted immunotherapy, suggesting sBCMA as a new biomarker for monitoring response to MM therapy [63]. Interestingly, unlike sBCMA, many studies have shown that the level of cell-surface BCMA does not seem to affect the response to BCMA-targeted immunotherapy [64]. It is quite possible that the varied levels of surface BCMA in MM patients are simply the result of the shedding variations of the membrane BCMA. High level of sBCMA may interfere with BCMA-targeted immunotherapy by reducing the total amount of cell-surface BCMA and sequestering circulating ligands or anti-BCMA antibodies, thereby inhibiting efficient binding to MM cells [65, 66]. It has been recently reported that a γ-secretase inhibitor (GSI, LY3039478/JSMD194) decreased sBCMA concentration, while increased cell-surface BCMA expression concurrently in MM cell lines, patient tumor cells, in murine models. This inhibitor significantly improved in vitro tumor recognition and in vivo anti-tumor efficacy of BCMA-specific chimeric antigen receptor (CAR)-T cells. Preclinical study has also discovered that short-term GSI administration to MM patients markedly increased the percentage of BCMA^+^ tumor cells [67]. Currently, a phase 1 clinical trial (NCT03502577) is ongoing to evaluate the safety and efficacy of combining CAR-T therapy with GSI, as well as cyclophosphamide (CTX), and fludarabine (FAMP) to treat patients with relapsed or persistent MM.

## BCMA-targeted immunotherapy

**Fig. 3:**
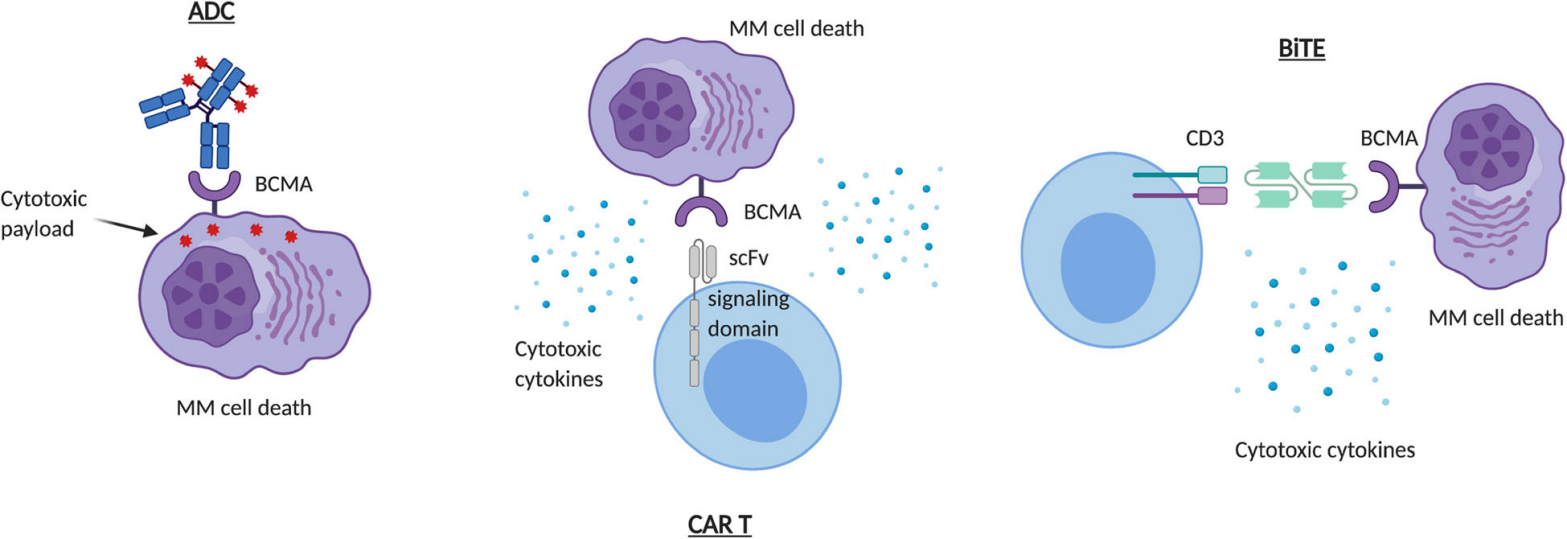
BCMA-targeted immunotherapies. **a** Antibody-drug conjugate (ADC). Upon binding to BCMA on the surface of multiple myeloma (MM) cells, ADC is internalized first and the linker is hydrolyzed inside of the lysosomes or endosomes, releasing the payloads that lead to cell death. **b** Chimeric antigen receptor (CAR)-T cells. The ectodomain of the BCMA scFv on the CAR-T cells binds to BCMA on the surface of MM cells. This leads to activation of the CAR-T cells which release cytotoxic cytokines and cause MM cell death. **c** Bispecific T cell engager (BiTE). The dual BCMA- and CD3-scFv-containing BiTE binds concomitantly to CD3 and BCMA, facilitating T cell/MM cell crosslinking, followed by CD4^+^/CD8^+^ T cell activation and secretion of cytotoxic cytokines, leading to MM cell death

Early studies on anti-BCMA antibodies showed robust cytotoxic activity against MM cells in vitro [68]. Currently, multiple innovative BCMA-targeted treatment modalities, including antibody-drug conjugate (ADC), CAR-T cells, and bispecific T cell engager (BiTE), are under active clinical development [13, 14, 69–77] (Fig. 3).

## BCMA antibody-drug conjugate (ADC)

ADC composes of three essential components: a mAb that recognizes a tumor-specific antigen, a cytotoxic molecule often referred to as payload, and a chemical linker that connects the mAb and payload [78–81]. Upon binding to the corresponding antigen on the surface of tumor cells, ADC is internalized first and the linker is hydrolyzed inside of the lysosomes or endosomes, releasing the payloads that lead to cell death by damaging DNA or impeding microtubule assembly. ADC becomes attractive because it enhances targeted killing of tumors while sparing normal tissues, thereby minimizing toxicity. With the improvement of engineering technology, the newest generation of ADCs can be generated through site-specific conjugation and has homogenous drug-antibody ratio as well as better stability in circulation [80–82]. Multiple ADCs have been approved for clinical therapy of lymphoma and leukemia [79–81, 83, 84]. Multiple BCMA ADCs are under clinical development (Table 1).

**Table 1:** BCMA-targeted antibody-drug conjugates in clinical trials

Name	Structure	Clinical trial information	Inclusion/exclusion criteria	Pt characteristics	Dosage	Major response	Most common AE
Belantamab mafodotin **(**GSK2857916)	Linker: non-cleavable MC Payload: MMAF	Phase 1 NCT02064387, DREAMM-1 [74, 85]	R/R MM received or were refractory to ASCT, alkylators, PI, and IMiD	35 pts in dose expansion phase; median age 60; high-risk cytogenetics 13 (37%); 14 (40%) pts received > 5 prior lines; mDOF: 12.5 mo	3.4 mg/kg every 3 wks	ORR 60%; sCR 2 (6%), CR 3 (9%), VGPR 14 (40%); mPFS 12 mo; mDOR 14.3 mo	G3+ thrombocytopenia (35%) anemia (17%); G1,2 corneal events: blurry vision (52%), dry eyes (37%),
		Phase 2 NCT03525678, DREAMM-2 [70]	R/R MM received or were refractory to ≥ 3 anti-MM therapies, including ASCT, alkylators, PI, IMiD, and CD38 mAb	196 pts − 2.5 mg/kg cohort 97 pts; median age 65; high-risk cytogenetics 41 (42%); median prior therapies 7; mDOF 6.3 mo − 3.4 mg/kg cohort: 99 pts; median age 67; high-risk cytogenetics 47 (47%); median prior therapies 6; mDOF 6.9 mo	2.5 or 3.4 mg/kg every 3 wks	− 2.5 mg/kg cohort: ORR 30 (31%); sCR/CR 3 (3%), VGPR 15 (15%); PD 56 (58%); mPFS 2.9 mo; − 3.4 mg/kg cohort: ORR 34 (34%); sCR/CR 3 (3%), VGPR 17 (17%); PD 55 (56%); mPFS 4.9 mo;	G3+ keratopathy (27% in the 2.5 mg/kg cohort and 21% in the 3.4 mg/kg cohort), thrombocytopenia (20% and 33%), and anemia (20% and 25%). TRD 2
MEDI2228	Linker: protease-cleavable Payload: PBD	Phase 1 NCT03489525,	R/R MM received or were refractory to all standard therapy including PI, IMiD, and ASCT	–	–	–	–
HDP-101	Linker: non-cleavable MC Payload: Amanitin	Preclinical	–	–	–	–	–

*AE* adverse event, *CR* complete response, *G* grade, *mDOF* median duration of follow-up, *MC* maleimidocaproyl, *MMAF* monomethyl auristatin F, *mo* month, *mDOR* median duration of response, *mPFS* median progression-free survival, *ORR* overall response rate, *PBD* pyrrolobenzodiazepine, *PD* progressive disease, *pt* patient, *sCR* stringent complete response, *VGPR* very good partial response, *wk* week

### Belantamab mafodotin (GSK2857916; Blenrep; GlaxoSmithKline, GSK)

Belantamab mafodotin (GSK2857916) is a humanized anti-BCMA IgG1 mAb conjugated to monomethyl auristatin F (MMAF, mafodotin) via a non-cleavable maleimidocaproyl (MC) linker. Mafodotin is released after complete proteolytic degradation of the whole mAb backbone as well as the linker by lysosomes. It then binds to tubulin and impedes microtubule assembly, leading to G2/M cell cycle arrest and subsequent cell apoptosis [80, 86]. Preclinical study discovered that GSK2857916 markedly inhibited BCMA+ MM cell growth in a dose- and time-dependent manner, with low bystander toxicity on surrounding BCMA− cells. It also rapidly eliminated MM cells in subcutaneous and systemic MM mouse models, leading to tumor-free duration up to 3.5 months. Another study of GSK2857916 demonstrated strong ADCC activity which was further enhanced by lenalidomide [33].

A multicenter, open-label, first-in-human phase 1 trial (DREAMM-1, NCT02064387) recruited 73 adult patients with R/R MM who failed autologous or allogenic stem cell transplantation (ASCT), alkylators, PIs, and IMiDs. In the dose-escalation phase, 38 patients received GSK2857916 (0.03–4.60 mg/kg) through 1-h intravenous infusions once every 3 weeks. No dose-limiting toxicities (DLT) or maximum tolerated dose (MTD) were identified. 3.40 mg/kg every 3 weeks was selected as the recommended dose for the dose-expansion phase of study which enrolled 35 patients [74]. According to a recent update, 21 patients showed responses with an overall response rate (ORR) of 60%. Two (6%) patients achieved stringent complete response (sCR), 3 (9%) patients achieved complete response (CR), and 14 (40%) patients experienced very good partial response (VGPR). The median progression-free survival (mPFS) and median duration of response (mDOR) were 12 and 14.3 months, respectively. There were mostly grade 1–2 adverse events (AEs), with thrombocytopenia (35%) and anemia (17%) being the most common treatment-related serious AEs (SAEs; grade ≥ 3 AEs). Grade 1–2 corneal events such as blurry vision (52%), dry eyes (37%), and photophobia (29%) were also frequently encountered. No cases of treatment-related death have been reported so far [85]. The response rate again did not appear to be associated with the expression level of cell-surface BCMA. However, non-responders were found to have a higher baseline level of circulating sBCMA than the responders. A higher dose of GSK2857916 (≥ 1.92 mg/kg) was required to bind a large fraction of sBCMA [64].

A multicenter randomized phase 2 trial (DREAMM-2, NCT03525678) is further evaluating the safety and efficacy of belantamab mafodotin (BLMF) in R/R MM [70]. There were 196 evaluable adults with R/R MM who had disease progression after receiving ≥ 3 previous lines of anti-MM treatments, were refractory to PIs or IMiDs, were refractory or intolerant to an anti-CD38 mAb, and had undergone ASCT or were ineligible for transplant. These patients were randomized to receive either 2.5 mg/kg (*n* = 97) or 3.4 mg/kg (*n* = 99) of BLMF once every 3 weeks over 30 min of IV infusion. In the published report, the ORR was 31% for the patients in the 2.5 mg/kg cohort and 34% in the 3.4 mg/kg cohort. The most common severe adverse events were keratopathy, thrombocytopenia, and anemia. There were two deaths potentially related to the BLMF therapy (one case of sepsis and one case of hemophagocytic lymphohistiocytosis). Belantamab mafodotin (Blenrep, GSK) was recently approved by FDA for the therapy of highly refractory MM.

Several other phase 1, 2, or 3 trials are currently recruiting to investigate different combination regimens incorporating BLMF in patients with R/R MM, such as BLMF + pomalidomide + dexamethasone (DREAMM-3, NCT03715478) [87]; BLMF + pembrolizumab (antiPD-L1) (DREAMM-4, NCT03848845); BLMF + dexamethasone + lenalidomide (arm A); or bortezomib (arm B) (DREAMM-6, NCT03544281) [80]. Another phase 1/2 trial (DREAMM-5, NCT04126200) was planned to explore the synergistic effects combining BLMF with other novel anti-cancer agents, such as the T cell activating checkpoint mAbs: GSK3359609 (an IgG4 inducible T cell co-stimulatory agonist antibody that is Fc optimized to selectively enhance T cell function to enable anti-tumor responses), GSK3174998 (a humanized wild-type IgG1 anti-OX40 agonistic mAb), and PF-03084014 (a γ-secretase inhibitor) [88]. Recently, a phase 3 trial started recruiting to compare the efficacy and safety of BLMF to daratumumab in the combination regimen with bortezomib and dexamethasone (DREAMM-7, NCT04246047) [70].

### HDP-101

HDP-101 is a fully humanized mAb conjugated to amanitin via a non-cleavable MC linker. Amanitin is a DNA-toxic agent that impedes the transcription process by binding to eukaryotic RNA polymerase I at very low concentrations irrespective of the proliferation status of the target cells. It appears to be superior to microtubule inhibitors which mainly target proliferating cells [89]. Preclinical study discovered that when administrated at pico- to nanomolar concentrations, HDP-101 exhibited profound cytotoxicity to BCMA^+^ myeloma cell lines and non-proliferating primary MM cells isolated from patients with R/R MM irrespective of BCMA expression level [90, 91]. Dose-dependent tumor regression after HDP-101 treatment was also observed in mouse xenograft models with both subcutaneous and systemic MM. In vivo study also showed a favorable safety profile in non-human primates that mainly consisted of transient, mild to moderate increase in liver enzymes and lactate dehydrogenase. HDP-101 is yet to be advanced to in-human clinical trials [91].

### MEDI2228

MEDI2228 is a fully human antibody site-specifically conjugated to pyrrolobenzodiazepine (PBD) dimer via a protease-cleavable linker. PBD dimer is also a DNA-toxic agent that binds to the minor-groove and crosslinks DNA, leading to DNA damage and apoptotic cell death. Preclinical study demonstrated that MEDI2228 exhibited profound anti-tumor activity against different cell lines of MM and plasma cell leukemia (PCL), including cells resistant to lenalidomide, regardless of the level of cell-surface BCMA expression. A single injection of MEDI2228 at doses as low as 0.1 mg/kg induced tumor regression in various MM xenograft models. In vitro study also reported that the anti-tumor activity persisted even in the presence of high concentration of sBCMA because the antibody component of MEDI2228 possessed strong binding to membrane-bound BCMA but weak affinity to monomeric human BCMA [92]. New reports suggested that MEDI2228 might synergize with anti-CD38 mAbs or bortezomib to enhance anti-myeloma effect [93, 94]. A dose-escalation phase 1 clinical trial (NCT03489525) is currently evaluating the pharmacokinetics, safety, and tolerability of MEDI2228 in patients with R/R MM.

## BCMA-targeted CAR-T cells

CAR-T cell therapy is a novel immunotherapy that combines the advantage of target specificity of mAbs and cytotoxicity of T cells [12, 14, 95]. Molecular engineering of new generation of CARs makes CAR-T therapy more promising [69, 96–102]. Two CD19-engineered CAR-T cell products, axicabtagen ciloleucel (yescarta, Kite) and tisagenlecleucel (kymriah, Novartis), have been approved by the U.S. Food and Drug Administration (FDA) for therapy of advanced B cell malignancies [95]. Currently, more than 10 BCMA-targeted CAR-T products have entered early-phase clinical studies (Tables 2 and 3) [13, 14, 96]. Most of them showed high response rates, even in patients with high-risk features such as high-risk cytogenetics and extra-medullary disease at time of CAR-T cell infusion [115]. Cytokine release syndrome (CRS) and neurotoxicity (NTX) are the common CAR-T-related adverse events [116–122]. However, a significant portion of responders eventually relapsed. Larger studies with longer follow-up investigating the association between response and survival are still warranted [123].

**Table 2:** BCMA-targeted CAR-T cell products in clinical trials

Name	Manufacturer	BCMA scFv	Co-stimulatory	Transduction	Extra safety domain
CAR-BCMA	NCI	Murine	CD28	γ-Retrovirus	No
Idecabtagene vicleucel; Bb2121	Bluebird Bio and Celgene	Murine	4-1BB	Lentivirus	No
Bb21217	Bluebird Bio and Celgene	Murine	4-1BB	Lentivirus	Yes, PI3K inhibitor
LCAR-B38M	Nanjing Legend/Genscript Biotech	Bi-epitope	4-1BB	Lentivirus	No
JNJ-4528	Janssen Research & Development	Bi-epitope	4-1BB	Lentivirus	No
CT053	CARsgen Therapeutics	Fully human	4-1BB	Lentivirus	No
P-BCMA-101	Poseida Therapeutics	Fully human anti-BCMA Centyrin^TM^	4-1BB	PiggyBac™ (PB) DNA Modification System	No
CART-BCMA	UPenn/Novartis	Fully human	4-1BB	Lentivirus	No
CT103A	Nanjing laso Biotherapeutics	Fully human	4-1BB	Lentivirus	No
JCARH125	Juno Therapeutics	Fully human	4-1BB	Lentivirus	No
MCARH171	MSKCC	Fully human	4-1BB	γ-Retrovirus	Yes, tEGFR
BCMA CAR-T	HRAIN Biotech	Fully human	4-1BB	γ-Retrovirus	Yes, tEGFR
KITE-585	Kite, Gilead company	Fully human	CD28	Lentivirus	No

**Table 3:** Interim results of clinical trials of BCMA-targeted CAR-T cell products

Name	Clinical trial information	Inclusion/exclusion criteria	Pt characteristics	Pre-condition	Dosage	Pharmacokinetics	Major response	Most common AE
Idecabtagene vicleucel; Bb2121	Phase 1b NCT02658929 [71]	R/R MM who received or were refractory to ≥ 3 prior lines, including PI, IMiD	33 pts (21 in dose-escalation; 12 in dose expansion); median age 60; median prior lines 7; high-risk cytogenetics 13 (45%); mDOF 11.3 mo	CTX + FAMP	150 or 450 × 10^6^ cells/pt	Expansion at all dose level, persist up to 1 yr	ORR 85%; CR 15 (45%); mPFS 11.8 mo	G3+ neutropenia (85%), leukopenia (58%), thrombocytopenia (45%), anemia (45%); **CRS** 23 (70%) G1–2, 2 (6%) G3+; **NTX** 14 (42%) G1–2, 13 (39%) G3+
	Phase 2 NCT0336174 [103]	R/R MM who received or were refractory to ≥ 3 prior lines, including PI, IMiD, and CD38 mAb	128 pts (54 received 450 × 10^6^ cells); median age 61; median prior lines 6; triple-refractory 108 (84%); penta-refractory 33 (26%); mDOF 11.3 mo	CTX + FAMP	150–450 × 10^6^ cells/pt	Peak on d11, detectable in 29/49 (59%) pts at 6 mo and 4/11 (36%) pts at 12 mo	ORR 73.4%; CR 31.3%; mPFS 11.3 mo 450 × 10^6^ cells dose cohort: ORR 81.5%; CR 35.2%; mPFS 11.3 mo	All grades cytopenia (94%); **CRS** 107 (83.6%) G1–2, 7 (5.5%) G3+; **NTX** 19 (14.9%) G1–2, 4 (3.1%) G3+
Bb21217	Phase 1 NCT03274219 [104]	R/R MM who received or were refractory to ≥ 3 prior lines, including PI, IMiD; ≥ 50% cell-surface BCMA expression	22 pts; median age 63; median prior lines 7; ASCT 18 (82%); high-risk cytogenetics 7 (32%); mDOF 23 wk	CTX + FAMP	150 or 350 or 450 × 10^6^ cells/pt	6/8 detectable at 6 mo, 2/2 detectable at 12 mo	ORR 83%; PD 6	**CRS** 7 G1–2, 1 G3+; **NTX** 3 G1–2, 2 G3+
LCAR-B38M	Phase 1/2 NCT03090659 [105]	R/R MM who received or were refractory to ≥ 1 prior lines	57 pts; median age 54 median prior lines 3; ASCT 10 (18%); mDOF 19 mo	CTX	Avg 0.5 × 10^6^ cells/kg 3 split infusions	Detectable till 4 mo, at most 10 mo	ORR 88%; CR 42 (74%), VGPR 2 (4%), PR 6 (11%); mPFS 20 mo; 18-mo PFS 50%; 18-mo OS 68%	G3+ leukopenia (30%), thrombocytopenia (23%), increased AST (21%); **CRS** 46 (82%) G1–2, 4 (7%) G3+; **NTX** 1 (2%) G1–2
	Phase 1/2 NCT03090659 [106]	R/R MM who received or were refractory to ≥ 3 prior lines,	17 pts; median age 55 median prior lines 5; ASCT 8 (47%); mDOF 22 mo	CTX ± FAMP	Avg 0.7 × 10^6^ cells/kg 1 infusion or 3 split infusions	Peak on d6–30, detectable till up to 9 mo	ORR 88%; CR 14 (82%), VGPR 1 (4%); mPFS 12 mo; 1-yr PFS 52.9%; 1-yr OS 82.3%	G3+ cytopenia 10 (59%); G3+ liver toxicity 5 (29%); **CRS** 10 (59%) G1–2, 7 (41%) G3+; **NTX** 0
JNJ-4528	Phase 1b/2 NCT03548207 [107]	R/R MM who received or were refractory to ≥ 3 prior lines, including PI, IMiD, CD38 mAb	29 pts; median age 61; median prior lines 5; 76% penta-exposed, 86% triple-refractory, 31% penta-refractory; mDOF 9 mo	CTX + FAMP	Avg 0.75 × 10^6^ cells/kg	Peak on d10-14, detectable till 6 mo, memory CD8+ CAR-T	ORR 100%; sCR 22 (76%), VGPR 6 (21%), PR 1 (3%); 6-mo PFS 93%; best mPFS 15 mo	G3+ neutropenia (100%), thrombocytopenia (69%), leukopenia (59%); **CRS** 27 (93%) G1–2, 2 (9%) G3+; **NTX** 3 G1–2, 1 G3+; **DLT** 1; **TRD** 1
CT053	Phase 1 NCT03716856 [108]	R/R MM who received or were refractory to ≥ 2 prior lines	24 pts; median age 60 median prior lines 4.5 high-risk cytogenetics 9 (38%); mDOF 333 d	CTX + FAMP	1.5 × 10^8^ cells/pt	Peak on d7–21, detectable till 172d, at most 341d	ORR 87.5%; sCR 14 (71%), CR 5 (21%), VGPR 1 (4%); mPFS 281 d; PD 9	G3+ leukopenia (87.5%), neutropenia (66.7%), lymphopenia (79.2%), thrombocytopenia (25%); **CRS** 15 (62.5%) G1–2; **NTX** 2 (8%) G1–2, 1 (4%) G3+
P-BCMA-101	Phase 2 NCT03288493 [109]	R/R MM who received or were refractory to ≥ 3 prior lines, including PI, IMiD, CD38 mAb	12 pts; prior lines 3–9 mDOF 3 wk	CTX + FAMP	0.75–15 × 10^6^ cells/kg	Peak at 2–3 wks, remain detectable at 3 mo	6 pts in higher dose cohort: ORR 83%; 1 sCR 1 VGPR 3 PR	G3+ cytopenia and febrile neutropenia; **CRS** 1 (8%) G1–2; **NTX** 0
CART-BCMA	Phase 1b NCT02546167 [110]	R/R MM who received or were refractory to ≥ 3 prior lines, including PI, IMiD,	25 pts; median age 58; median prior lines 7; high-risk cytogenetics 24 (94%); ASCT 23 (92%); panta-refractory 11 (44%); mDOF 24 mo	None or CTX	1–5 × 10^7^ or 1–5 × 10^8^ cells/pt	Peak on d10–14, remain detectable at 6 mo	ORR 48%; CR 2 (8%), VGPR 5 (20%), PR 5 (20%); best mPFS 125 d; PD 22	G3+ leukopenia (44%), neutropenia (44%), lymphopenia (36%); **CRS** 14 (56%) G1–2, 8 (32%) G3+; **NTX** 5 (20%) G1–2, 3 (12%) G3+
CT103A	Phase 1 ChiCTR 1800018137 [111]	R/R MM who received or were refractory to ≥ 3 prior lines, including PI, IMiD,	16 pts; median prior lines 4; mDOF 195 d	CTX + FAMP	1–8 × 10^6^ cells/kg	Peak at 2 wks, remain detectable at 6 mo	ORR 100%; CR/sCR 12 (75%), VGPR 2 (12.5%)	**CRS** 15 (94%) G1–2, 1 (6%) G3+; **NTX** 0; **DLT** 1; **TRD** 1
JCARH125	Phase 1/2 NCT03430011 [112]	R/R MM who received or were refractory to ≥ 3 prior lines, including PI, IMiD, CD38 mAb, ASCT	8 pts; median age 53; median prior lines 10; ASCT 8 (88%); panta-refractory 4 (50%); mDOF 5 wk	CTX + FAMP	50 or 150 × 10^6^ cells/pt	–	ORR 100%; sCR/CR 3 (37.5%), VGPR 2 (25%), PR 2 (25%); PD 0	**CRS** 6 (75%) G1–2; **NTX** 2 (25%) G1–2, 1 (12.5%) G3+
MCARH171	Phase 1 NCT03070327 [113]	R/R MM who received or were refractory to ≥ 2 prior lines, including PI, IMiD	11 pts; median prior lines 6	CTX + FAMP	72, 137, 475, 818 × 10^6^ cells/pt 1 to 2 doses	Peak expansion found in dose cohort 475, 818 × 10^6^	ORR 64%; mDOR 106 d	**CRS** 4 (40%) G1–2, 2 (20%) G3+; **NTX** 1 (9%) G1–2
BCMA CAR-T	Phase 1 NCT03093168 [114]	R/R MM who received or were refractory to ≥ 2 prior lines, including PI, IMiD; ≥ 5% cell-surface BCMA	44 pts median prior lines ≥ 2 mDOF ≥ 1 mo	CTX + FAMP	9 × 10^6^ cells/kg	Expansion and persistence throughout the DOF	ORR 79.6%; sCR 2 (4.5%), CR 16 (36%), VGPR 8 (18%), PR 8 (18%); mPFS 15 mo; 2-yr PFS 49.16%; 2-yr OS 53.95%	**CRS** 10 (22.7%) G1–2, 3 (6.8%) G3+

*Avg* average, *ASCT* autologous or allogenic stem cell transplantation, *CR* complete response, *CRS* cytokine release syndrome, *CTX* cyclophosphamide, *d* day, *DLT* dose-limiting toxicity, *FAMP* fludarabine, *G* grade, *IMiD* immunomodulatory imide drugs, *mDOF* median duration of follow-up, *mDOR* median duration of response, *mo* month, *mPFS* median progression-free survival, *NTX* neurotoxicity, *ORR* overall response rate, *OS* overall survival, *PD* progressive disease, PI proteasome inhibitor, *PR* partial response, *pt* patient, *sCR* stringent complete response, *TRD* treatment-related death, *VGPR* very good partial response, *wk* week, *yr* year

### CAR-BCMA

One of the first anti-BCMA CAR-T cell products was developed through a γ-retroviral vector that encoded a second-generation CAR containing a murine BCMA scFv and a CD28 co-stimulatory domain. These engineered T cells gained the ability of BCMA recognition, cytokine production, proliferation, cytotoxicity, and in vivo tumor eradication [34].

The first-in-human dose-escalation phase 1 trial (NCT02215967) investigating the safety and efficacy of this CAR-BCMA cell therapy initially reported 12 patients with R/R MM [63]. Three doses of 300 mg/m^2^ CTX plus 30 mg/m^2^ FAMP were administered daily on days 5, 4, and 3 before a single dose of CAR-BCMA T cell infusion on day 0. Four dose levels of CAR-T cells were selected: 0.3, 1.0, 3.0, and 9.0 × 10^6^ cells/kg. Overall, 1 patient achieved sCR, 3 patients entered partial remission (PR), and 8 patients maintained stable disease (SD) for up to 16 weeks. Among the 6 patients receiving the 2 lowest dose levels, limited anti-myeloma activity and mild toxicity occurred. Better clinical responses were observed in the 2 higher dose groups. One patient treated on the third dose level obtained VGPR. Among the 2 patients treated on the fourth dose level, one patient achieved sCR for 17 weeks, with BM plasma cells eradicated after treatment, and the other patient underwent VGPR with BM plasma cells undetectable by flow cytometry 28 weeks after treatment and serum monoclonal protein decreased by more than 95%. However, patients receiving higher doses presented with more prolonged cytopenia and a higher risk of severe CRS. In addition, a remarkable decrease of sBCMA level after CAR-BCMA T cell infusion was observed in patients with the most impressive anti-myeloma responses [63]. 9.0 × 10^6^ cells/kg was selected for the dose expansion study in 16 patients who failed an average of 9.5 prior lines of MM. The ORR was 81%, with 63% patients achieving VGPR or CR. Median event-free survival (mEFS) was 31 weeks. In addition to eradication of BM myeloma cells, soft tissue plasmacytomas were also eliminated. Negative minimal residual disease (MRD) (≤ 10^−4^ nucleated cells) was achieved in the BM in 11 responders. Higher peak blood CAR+ cell levels were associated with better anti-tumor responses. BCMA antigen loss was also observed which might contribute to therapy resistance and disease relapse. CRS toxicities were substantial especially in patients with high MM burdens but were generally reversible. NTX was limited to confusion or delirium in the setting of severe CRS. Cytopenia was frequently encountered early after CAR-BCMA T cell infusion in most patients and was attributable to the CTX and FAMP conditioning chemotherapy. Some cases with prolonged cytopenia were also reversible with treatment [124].

### Idecabtagene vicleucel (ide-cel; bb2121) and bb21217

Bb2121 product consists of autologous T cells transduced with a lentiviral vector encoding a second-generation CAR which incorporates a murine anti-BCMA scFv, a 4-1BB co-stimulatory motif and a CD3ζ T cell activation domain. Preclinical data showed that bb2121 demonstrated robust in vitro killing of MM cells independent of the BCMA expression levels or the presence of sBCMA. A single administration of bb2121 resulted in rapid and sustained tumor elimination and 100% survival in a mouse model of human MM [125].

Based on these findings, a multicenter phase 1 trial (CRB-401, NCT02658929) was initiated in patients with R/R MM who had failed at least 3 (median 7) previous lines of therapy, including a PI, an IMiD, or are double-refractory. Doses of 50 × 10^6^, 150 × 10^6^, 450 × 10^6^, or 800 × 10^6^ total CAR+ T cells were tested in the dose-escalation phase, of which 150 × 10^6^ to 450 × 10^6^ total CAR+ T cells were selected for the dose-expansion phase. Results of the first 33 consecutive patients with an average of 11.3 months follow-up were reported recently. The ORR was 85% (45% CR). The mPFS was 11.8 months. Sixteen responders who could be evaluated for MRD all obtained MRD-negative status and were found to have better outcome with an mPFS of 17.7 months. Better clinical responses were found in high-dose cohorts (≥ 150 × 10^6^ total CAR+ cells) and in patients with high-risk cytogenetic profiles. Response rate appeared to be unrelated to the amount of tumor-surface BCMA. However, a greater depth of changes of sBCMA level after infusion was found to correlate with earlier and more durable responses. CAR-T cell expansion was observed at all dose levels and was associated with responses. CAR-T cells persisted up to 1 year after the infusion. Hematologic toxicity was the most common SAE. Non-hematologic toxic effects were primarily of grade 2 or lower. CRS was observed in 25 patients (76%), which was of grade 1 or 2 in 23 patients (70%) and grade 3 in 2 patients (6%). NTX occurred in 14 patients (42%) and were of grade 1 or 2 in 13 patients (39%) and grade 4 in 1 patient (3%) but was reversible. No cases of treatment-related death have been reported so far [71, 126, 127].

In view of the favorable safety profile and efficacy, a phase 2 clinical trial of bb2121 in patients with R/R MM (KarMMa, NCT03361748) has been started. Eligible participants are R/R MM patients who have received at least 3 prior lines of therapy including IMiD, PI, and CD38 mAb and were refractory to their last regimen. One dose of bb2121 (dose range: 150-450 × 10^6^ total CAR+ cells) is given after lymphodepletion [71, 128]. According to a recent update, as of October 16, 2019, 128 patients received the infusion (4 patients received 150 × 10^6^ total CAR+ cells, 70 patients received 300 × 10^6^ total CAR+ cells, and 54 patients received 450 × 10^6^ total CAR+ cells). Median number of prior therapies was 6. Median duration of follow-up (mDOF) was 11.3 months. Ninety-four patients (ORR 73.4%) showed responses, including 40 (31.3%) CR, and mPFS was 8.6 months. Those who received the dose of 450 × 10^6^ total CAR+ cells achieved 81.5% ORR, 35.2% CR, and mPFS was 11.3 months, indicating that higher doses might be associated to better response and outcomes. CAR-T cell expansion peaks at day 11, with durable persistence, as circulating CAR-T cells were detectable in 29/49 (59%) patients at 6 months and 4/11 (36%) patients at 12 months post-infusion. Higher expansion was also observed when high dose was given. Most common AE was cytopenia (97%). One hundred seven (83.6%) patients encountered CRS including 7 (5.5%) grade ≥ 3 events and 1 death. Twenty-three (18%) patients encountered NTX including 4 (3.1%) grade ≥ 3 events [103].

Another phase 2 study (KarMMa-2, NCT03601078) looking at patients with high-risk MM including those with early relapse or inadequate response post-ASCT has also been initiated. In addition, a multicenter, randomized, open-label, phase 3 trial is recruiting to compare the efficacy and safety of bb2121 versus standard triplet regimens (daratumumab + pomalidomide + dexamethasone, or daratumumab + bortezomib + dexamethasone, or ixazomib + lenalidomide + dexamethasone) in patients with R/R MM (KarMMa-3, NCT03651128).

Bb21217 is constructed on the basis of the bb2121 CAR structure with an extra domain of bb007 which encodes a Phosphoinositide 3-kinases (PI3K) inhibitor to enrich the CAR-T cells displaying a memory-like phenotype. It has been suggested that selected CAR-T cells with this phenotype might be more potent and more persistent than unselected CAR-T cells. A first-in-human, multicenter phase 1 dose-escalation trial (CRB-402, NCT03274219) is ongoing to assess the safety, pharmacokinetics, efficacy, and duration of bb21217 CAR-T cells in patients with R/R MM who received ≥ 3 prior regimens, including a PI and an IMiD, or are double-refractory to both classes. Only patients with ≥ 50% BCMA expression detected by immunohistochemistry (IHC) on malignant PCs were enrolled. Four dose levels were planned: 150 × 10^6^, 450 × 10^6^, or 800 × 10^6^, and 1200 × 10^6^ total CAR+ T cells [104]. According to the latest update, 22 patients (median age 63) have received bb21217 infusion so far (12 at 150 × 10^6^, 6 at 300 × 10^6^, and 4 at 450 × 10^6^). The median number of prior regimens received was 7. Eighteen patients (82%) had undergone ASCT, and 7 had high-risk cytogenetics. Eighteen patients were evaluable for initial (1 month) clinical responses. Among the 15 patients (83%) who responded, 6 have progressed. 10/10 evaluable responders obtained undetectable MRD status. 6/8 patients evaluable at 6 months and 2/2 patients evaluable at 12 months presented with detectable CAR+ T cells in peripheral blood. Fifteen patients (68%) developed manageable CRS (5 grade 1, 2 grade 2, 1 grade 3). Five patients (23%) developed NTX (1 grade 1, 2 grade 2, 1 grade 3, 1 grade 4 featured by encephalopathy); all were successfully resolved [128]. However, no evidence is available to yet show bb21217 is more advanced than bb2121 in terms of persistence of clinical response at present.

### LCAR-B38M (JNJ-4528)

LCAR-B38M, also known as JNJ-68284528 (JNJ-4528), is a bispecific second-generation CAR-T cell product directed against 2 distinct BCMA epitopes. This bi-epitope targeting significantly improves the binding avidity and distinguishes LCAR-B38M from all the other BCMA-targeted CAR-T products. An ongoing, single-arm, open-label, multicenter phase 1 trial (LEGEND-2, NCT03090659) of LCAR-B38M was initiated in patients with R/R MM in China. After achieving lymphodepletion with 3 doses of CTX 300 mg/m^2^, weight-adjusted LCAR-B38M CAR-T cells (median, 0.5 × 10^6^ cells/kg [range, 0.07 to 2.1 × 10^6^]) were administered in 3 infusions (20, 30, and 50% of total dose) over 7 days. As of December 31, 2018, 57 eligible patients (median age 54) who failed at least 1 (median 3) prior regimens (must at least have contained bortezomib) have received the therapy with a mDOF of 19 months [76]. The ORR was 88% with 74% CR, 2 patients (4%) obtained VGPR, and 6 patients (11%) obtained PR. 39/42 (93%) patients with CR achieved undetectable MRD status. The median time to initial response was 1.2 month. The 18-month OS was 68% with an mDOR of 22 months for all responders and 27 months for MRD-negative patients. The 18-month PFS was 50% with an mPFS of 20 months for all patients and 28 months for MRD-negative patients. The clinical response was found to be related to neither the LCAR-B38M CAR-T cell dose nor the level of BCMA expression. LCAR-B38M became undetectable in peripheral blood in majority of patients at 4 months; however, it persisted up to 10 months in 5 patients. The most common SAEs were leukopenia (30%), thrombocytopenia (23%), and increased aspartate aminotransferase (AST) (21%). CRS was mostly grades 1 and 2, with only 4 patients (7%) experiencing grade 3 toxicity, which were all reversible. The median time to CRS onset was 9 days post-infusion. No statistically significant correlation was found between CRS and the dose of therapy. NTX was reported in 1 patient who presented with grade 1 aphasia, agitation, and seizure-like activity [75, 76, 105, 129].

In a separate report (NCT03090659), LCAR-B38M was tested in 17 patients (median age 55) with R/R MM who failed at least 3 prior lines of therapy enrolled from three clinical centers in China different from the aforementioned study. Two different treatment protocols were adopted by different sites. In 2 clinical centers, 8 patients received a lymphodepletion regimen of CTX 250 mg/m^2^ + FAMP 25 mg/m^2^, intravenously daily for 3 days, and then CAR-T cells split into 3 infusions (day 0, 3, and 6) 5 days after lymphodepletion, while in 1 clinical center, 9 patients received CTX 300 mg/m^2^ intravenously daily for 3 days, and CAR-T cells were administered via only one infusion. The mean dose was 0.7 × 10^6^ (range, 0.2–1.5 × 10^6^) CAR-T cells/kg [106, 130]. As of July 20, 2019, the ORR was 88%. Fourteen patients (82%) achieved CR, and 1 patient (6%) underwent VGPR. All 14 CR patients obtained undetectable MRD status. The median time to first response was 1 month. At a mDOF of 22 months, 6 (38%) patients remain progression-free, with a mPFS of 12 months, 1-year PFS of 52.9%, and 1-year OS of 82.3%. Positive anti-LCAR-B38M antibody represented a risk factor for relapse or progressive disease (PD), while patients who underwent prior ASCT showed a more durable response. LCAR-B38M CAR-T cells peaked around day 6 to 30 and persisted in most patients up to 9 months. Regarding toxicities, grade ≥ 3 cytopenia occurred in 10 patients. All patients experienced grade 1–2 liver toxicity. CRS was observed in all patients. Ten patients experienced grade 1 or 2 CRS, and 7 patients had grade ≥ 3 CRS after infusion. One patient died of the concurrence of severe CRS and tumor lysis syndrome (TLS) despite the administration of hemodialysis, tocilizumab, etanercept, and other supportive care. The study pointed out several potential indicators for severe CRS such as the abundance of BCMA, cytogenetic marker del(17p), and the elevation of interleukin-6 (IL-6) [76, 130].

A U.S. phase 1b/2 trial (CARTITUDE-1, NCT03548207) is ongoing to assess the safety and efficacy of JNJ-4528 (LCAR-B38M), in patients with R/R MM who received ≥ 3 prior regimens or were double-refractory to a PI and IMiD, and received anti-CD38 antibody. A single infusion of JNJ-4528 at the targeted dose of 0.75 × 10^6^ CAR+ cells/kg (range 0.5–1.0 × 10^6^) was administered 5–7 days after initiation of the standard conditioning regimen with CTX and FAMP. According to a recent update, 29 patients (median age 61) who failed a median number of 5 prior lines entered the phase 1b portion of the study with a mDOF of 9 months. An ORR of 100% has been found, with 22 (76%) sCRs, 6 (21%) VGPRs, and 1 (3%) partial response (PR). Median time to ≥ CR was 2 months. The response rate was independent of baseline BCMA expression. MRD negativity at 10^−5^ or 10^−6^ in the BM was achieved in all evaluable patients at 6 months. At the time of data cutoff, 26/29 patients are progression-free, with 6-month progression-free survival rate of 93%, and longest response ongoing at 15-month follow-up. Cytopenia was the most common SAE. Twenty-seven patients (93%) experienced grade 1 to 2 CRS, with 1 grade 3 case and 1 grade 5 event which became the DLT. Median time to CRS onset was 7 days post-infusion. Most CRS events lasted around 4 days and were manageable with support, tocilizumab, or corticosteroid therapy. Four patients experienced NTX with 3 grade 1 to 2 and 1 grade 3. One death related to grade 5 CRS and 1 non-treatment-related death (acute myeloid leukemia) were reported [107, 131]. Majority of patients reached the peak expansion of JNJ-4528 CAR+ cellular and transgene levels around 10–14 days post-infusion with a concurrent decline of sBCMA levels. JNJ-4528 CAR-T cells remained detectable in 22/28 patients at 6-month follow-up. At peak expansion, there was a preferential expansion of CD8+ CAR-T cells displaying predominantly a central memory (Tcm) phenotype (CCR7+ CD45RO+), which contributed to the high anti-tumor activity of JNJ-4528 at a relatively low dose. Serum cytokine levels also increased with the expansion of CAR-T cells. And increases in IL-6 correlated to the onset of CRS which was around day 7, similar to previous studies [107, 132]. In consistent with LEGEND-2, CARTITUDE-1 further confirmed the favorable efficacy and manageable toxicity profile of LCAR-B38M/JNJ-4528 CAR-T cell therapy. 0.75 × 10^6^ CAR+ cells/kg was the recommended phase 2 dose for future development.

Currently, phase 2 investigation of LCAR-B38M/JNJ-4528 CAR-T cell in patients with R/R MM has been initiated both in China (CARTIFAN-1, NCT03758417) and in the USA (CARTITUDE-2, NCT04133636). A phase 3 clinical trial is being planned to compare the efficacy and safety of JNJ-4528 to those of the standard therapy (PVd: Pomalidomide + Bortezomib + Dexamethasone; or DPd: Daratumumab + Pomalidomide + Dexamethasone) in patients with R/R MM (CARTITUDE-4, NCT04181827).

### CT053

CT053 is another CAR-T cell product in development in China. It is a second-generation CAR incorporating a fully human anti-BCMA scFv which is much less immunogenic, a 4-1BB co-stimulatory motif and a CD3ζ activation domain. CT053 is being investigated in multiple phase 1 trials in China (NCT03716856, NCT03302403, NCT03380039, and NCT03975907) that recruited patients with R/R MM who failed at least 2 (average 4.5) prior regimens. Participants received one cycle of CT053 CAR-BCMA after FAMP/CTX conditioning. As of June 30, 2019, 24 eligible patients (median age 60) have been enrolled. Most of them received a single dose of 1.5 × 10^8^ total CAR-T cells except 3 subjects who received 0.5 × 10^8^, 1 × 10^8^, and 1.8 × 10^8^ cells, respectively. The ORR was 87.5% (21/24) including 14 sCR, 5 CR, 1 VGPR, and 1 PR. 17 out of 20 evaluable patients achieved MRD-negative status. Efficacy was observed even at the lowest dose (0.5 × 10^8^) in a patient who maintained VGPR for 378+ days and achieved CR on day 437 and then sCR on day 502. mDOF was 333 days. Thirteen patients maintained CR/sCR. Nine patients have progressed so far with a mPFS of 281 days, 3 of whom died at the data cutoff date. Median T cell persistence was 172 days, and the longest was 341 days. Hematologic toxicity remained the most common treatment-related SAE. Fifteen of 24 patients (62.5%) experienced CRS, and all were low grades and recovered within 2–8 days. NTX was reported in 3 patients (12.5%) (2 grade 1, 1 reversible grade 3). No DLT has been observed at the time of analysis [108, 133]. Another multicenter phase 1b trial (NCT03915184) has started recruiting to evaluate the safety and efficacy of CT053 in R/R MM in the USA.

### P-BCMA-101

P-BCMA-101 is a novel second-generation CAR-T product that incorporates a fully human anti-BCMA Centyrin^TM^, 4-1BB co-stimulatory motif, and a CD3ζ activation domain. Centyrin is a fully human protein that presents with high specificity, binding affinity, more stability, and less immunogenicity. What makes it different from most CAR-T products is that P-BCMA-101 is produced using the piggyBac™ (PB) DNA Modification System instead of a viral vector [134]. Since the product does not depend on viral transfection, it may be more cost-effective. In addition, P-BCMA-101 contains a purified population of CAR+ cells with a high percentage of favorable stem cell memory T phenotype (T_SCM_). Preclinical data demonstrated prominent efficacy in reducing tumor burden and preventing recurrence in various xenograft models with a single dose of P-BCMA-101 [135]. A phase 1 dose-escalation trial (NCT03288493) has enrolled 12 patients with heavily pretreated MM (≥ 3 [3–9] prior lines, including a PI and an IMiD, or double-refractory). These patients received one dose of P-BCMA-101 ranging from 0.75 × 10^6^ to 15 × 10^6^ cells/kg after being conditioned by CTX and FAMP. Of the 6 patients who received higher dose of therapy, an ORR of 83%, with 1 sCR, 1 VGPR, and 3 PR, had been achieved within 2 weeks. The response rate did not correlate to the level of BCMA expression. P-BCMA-101 cell level peaked at 2–3 weeks and remained detectable at the data cutoff point as far as 3 months. Cytopenia and febrile neutropenia were still the most common SAEs. Of note, there was a minimal rate of CRS and NTX. Only 1 case of maximum grade 2 CRS was identified. Therefore, this novel CAR-T agent might generate an improved therapeutic index [109].

Based on the data of phase 1, a pivotal phase 2 study (PRIME; NCT03288493) has been designed. The trial plans to enroll 100 adult R/R MM patients who have failed at least 3 prior lines of therapy, including a PI, an IMiD, and CD38 targeted therapy. No pre-specified level of BCMA expression is required. Of note, patients getting prior CAR-T cells or BCMA-targeted agents are also eligible for the study. A single intravenous dose of P-BCMA-101 CAR-T cells (6–15 × 10^6^ cells/kg) will be administered after a standard 3-day CTX/FAMP conditioning regimen. Different from other CAR-T products, given the safety profile demonstrated during phase 1, no hospital admission is required and patients may receive P-BCMA-101 in an outpatient setting [136].

### CART-BCMA

Another lentiviral-transduced CAR-T cell product, CART-BCMA, contains a fully human BCMA-specific scFv with CD3ζ and 4-1BB signaling domains. Preclinical study demonstrated high specificity for BCMA and robust anti-myeloma activity [137]. CART-BCMA has been evaluated in a phase 1b study (NCT02546167) in R/R MM. Twenty-five patients (median age 58) refractory to a median number of 7 prior lines were enrolled and randomized into 3 different treatment cohorts: (1) 1 to 5 × 10^8^ CART-BCMA cells alone, (2) CTX 1.5 g/m^2^ + 1 to 5 × 10^7^ CART-BCMA cells, and (3) CTX 1.5 g/m^2^ + 1 to 5 × 10^8^ CART-BCMA cells. Responses were seen in all cohorts with or without lymphodepletion with ORR of 44% (4/9) in cohort 1, 20% (1/5) in cohort 2, and 64% (7/11) in cohort 3, including a total of 2 CR, 5 VGPR, and 5 PR. By the time of data cutoff, 3 patients remained progression-free. The mPFS was 65 days in cohort 1, 57 days in cohort 2, and 125 days in cohort 3. The study also discovered that better responses and CART-BCMA expansion were associated with higher CD4/CD8 T cell ratio and percentage of CD45RO-CD27+CD8+ T cells in the initial leukapheresis product. BCMA expression level was found to be decreased on residual MM cells of responders and increased in most patients with PD. Circulating CAR-T cell expansion generally peaked on days 10–14, remained detectable at 6 months in 14 of 17 (82%) patients tested, and persisted till 2.5 years after infusion in one patient with sCR. Hematologic toxicities were the most common SAE: leukopenia (44%), neutropenia (44%), and lymphopenia (36%). Grade 3–4 CRS and NTX were reported in 8 (32%) and 3 (12%) participants, respectively, thus making this product seemingly more toxic than other products. One patient died of candidemia and progressive MM following treatment for severe CRS and encephalopathy [110].

### CT103A

CT103A is a novel BCMA-targeting CAR-T cell product developed with a lentiviral vector encoding a second-generation CAR structure with a fully human scFv, 4-1BB co-stimulatory, and CD3ζ activation domain. It is currently undergoing a single-center phase 1 investigation in China (ChiCTR1800018137). As of August 1, 2019, 16 patients with R/R MM refractory to a median number of 4 prior lines have been enrolled. All patients received CT103A in a dose-escalation trial (four doses at 1, 3, 6, 8 × 10^6^/kg) after a CTX/FAMP conditioning regimen and were followed for an average of 195 days after infusion. The study showed an excellent response with an ORR of 100%, including 12 CR/sCR and 2 VGPR with 6 patients achieving CR/sCR within 2 weeks post-infusion. All 15 evaluable patients reached MRD-negative status. Circulating CT103A cells peaked at 2 weeks post-infusion and remained detectable in 12/16 patients at 6 months. All 16 patients developed low-grade CRS, with 1 grade 4 event at the dose of 6 × 10^6^/kg which was considered as a DLT. No NTX was observed. One patient died of lung infection at day 19 post-infusion [111, 138]. Despite this encouraging preliminary result, further studies are still warranted to evaluate the safety and efficacy of this fully human BCMA-targeted CT103A.

### JCARH125

JCARH125 is second-generation BCMA-targeted CAR-T product containing a fully human scFv, optimized spacer, 4-1BB co-stimulatory, and CD3ζ activation domain. Preclinical study demonstrated robust anti-tumor activity of JCARH125 against BCMA expressing cells as well as various xenograft models regardless of the antigen densities or the presence of sBCMA. Off-target activity was not observed [139]. A phase 1/2 multicenter trial (EVOLVE, NCT03430011) is investigating the safety and efficacy of JCARH125 in patients with high R/R MM, who have received ≥ 3 prior regimens, including ASCT, a PI, IMiD, and an anti-CD38 mAb, unless not a candidate. The first 2 dose levels at 50 and 150 × 10^6^ CAR+ T cells have been studied. As of July 12, 2018, all 8 evaluable patients out of 19 enrolled (median age 53; median number of prior lines 10) showed objective response, with 2 sCR, 1 CR, 2 VGPR, 2 PR, and 1 mild response (MR). The mDOF was 5 weeks. No PD was identified at the cutoff date. Six patients experienced grade 1 or 2 CRS, and 3 patients experienced NTX with 1 grade 3 lethargy resolved within 24 h after receiving steroids [112]. This study is still ongoing.

### MCARH171

MCARH171 is a second-generation CAR-T cell product incorporating a human BCMA scFv and a 4-1BB co-stimulatory motif, as well as a truncated epidermal growth factor receptor (tEGFR) safety system. Preclinical studies observed rapid in vivo expansion of CAR-T cells, eradication of large tumor burden, and durable protection to tumor re-challenge [140]. A dose-escalation phase 1 trial (NCT03070327) is currently evaluating the safety and efficacy of MCARH171 in patients with R/R MM. In this trial, participants received conditioning regimen with FAMP and CTX followed by MCARH171 infusion in 1–2 divided doses. Four dose levels were tested: 72 × 10^6^, 137 × 10^6^, 475 × 10^6^, 818 × 10^6^ total CAR+ T cells. As of July 16, 2018, 11 patients who failed an average of 6 prior myeloma regimens have received the therapy. The ORR was 64%, and the mDOR was 106 days. High-dose cohorts demonstrated higher peak expansion and longer persistence of MCARH171 as well as more durable clinical responses than low-dose cohorts. CRS occurred in 6 patients (4 grade 1–2, 2 grade 3). One patient experienced grade 2 encephalopathy which resolved within 24 h. No DLT was found [113].

## BCMA CAR-T cells with tEGFR

A Chinese company (Hrain Biotechnology) has also developed a second-generation γ-retrovirus-mediated anti-BCMA CAR-that contains a fully human scFv, 4-1BB co-stimulatory, CD3ζ activation domain along with the tEGFR safety system to facilitate tracking of the BCMA-targeting CAR-T cells. A phase 1 trial (NCT03093168) has been launched to evaluate the safety and efficacy of this BCMA CAR-T cell product for treating R/R MM. This study enrolled patients who failed at least 2 prior treatment regimens and have over 5% BCMA expression on PCs. A single dose of 9 × 10^6^ CAR^+^ cells/kg CAR-T cells were administered following the standard CTX/FAMP lymphodepletion regimen. As of March 1, 2019, 44 enrolled evaluable patients achieved an ORR of 79.6%, including 2 sCR, 16 CR, 8 VGPR, and 8 PR, and 16 patients reached MRD-negative status. At the data cutoff, mPFS was 15 months. The 24-months PFS and OS were 49.16% and 53.95%, respectively. Grade 1–2 CRS was identified in 10 (22.7%) patients, and 3 (6.8%) patients underwent manageable grade 3 CRS events. The study has been ongoing for more than 26 months so far [114].

### KITE-585

KITE-585 is a second-generation lentiviral-transduced CAR-T cell product which has a fully human anti-BCMA scFv, a CD28 co-stimulatory domain, and a CD3ζ activation domain. KITE-585 demonstrated potent in vitro and in vivo activity against MM cell lines even in the presence of soluble BCMA and also eradicated xenografted MM tumors in mice [141, 142]. A first-in-human, open-label, multicenter phase 1 study (NCT03318861) has been planned to evaluate the safety and feasibility of KITE-585 in R/R MM patients [143]. However, development of this product has subsequently been terminated.

## Bispecific CAR-T cells

Bispecific and multi-antigen-targeted CAR-T cells are under active development [97, 144]. Bispecific CAR-T cells targeting BCMA and another tumor-specific antigen such as CD19 and CD38 have recently been developed to improve clinical efficacy by reducing the risk of relapse due to antigen escape.

### CD19/BCMA bispecific CAR-T cells

Even though CD19 expression is lost in normal plasma cells [145, 146], CD19 is found to be expressed in a minute subset of less differentiated myeloma clones and CD19-targeted CAR-T cell therapy is effective in certain MM patients [147–150]. CD19+/BCMA- MM cells with enhanced clonogenic potential might contribute to the relapse after anti-BCMA CAR-T cell therapy [151]. It has been reported that combined infusion of both anti-CD19 and anti-BCMA CAR-T cells was feasible and produced promising responses with manageable toxicities in patients with R/R MM [152]. However, the addition of anti-CD19 CAR-T did not clearly prevent progression after anti-BCMA CAR-T therapy according the interim result of an ongoing study [153]. A BCMA-CD19 bispecific CAR-T product was recently manufactured in China. It was constructed by linking BCMA and CD19 scFv, joined by a CD8 hinge, transmembrane domain, co-stimulatory domain, and CD3ζ [154]. In preclinical study, BCMA-CD19 CAR-T cells were shown to be highly effective in eliminating MM tumor cells both in vitro and in vivo and appeared more cytotoxic than single CAR-T. A first-in-human trial has been initiated. Five patients with R/R MM were evaluated between 15 and 59 days post-CAR-T infusion. All patients showed responses within this short period of evaluation time, with 1 sCR, 3 VGPR, and 1 PR. The sCR patient maintained the status at the time or report (129 days). The CAR-T proliferation peak was reached on day 10. Three patients experienced grade 1 CRS. No SAEs were reported [154].

### BM38: CD38/BCMA bispecific CAR-T cells

CD38 is a glycoprotein expressed on PCs and other lymphoid cell populations. Due to its high and uniform expression on myeloma cells, CD38 is an ideal target for novel therapeutic strategies especially mAbs (daratumumab and isatuximab) [155–157]. BM38, a bispecific CAR-T cell product targeting BCMA and CD38, incorporates the anti-CD38 and anti-BCMA scFv in tandem plus 4-1BB signaling and CD3ζ domains. A phase 1 dose-escalating trial has been launched in China (ChiCTR1800018143). As of 31 July 2019, 16 patients with R/R MM who had received 2 prior treatment regimens received BM38 CAR-T cell infusion (dose range 0.5, 1.0, 2.0, 3.0, and 4.0 × 10^6^ cells/kg). mDOF was 36 weeks. Fourteen (87.5%) patients achieved ORR with 8 sCR, 2 VGPR, and 4 PR. Fourteen (87.5%) patients reached MRD-negative status, and all 5 (100%) extra-medullary lesions were eliminated. The longest duration of sCR was over 51 weeks. Five out of the 8 sCR patients maintained sCR status, 2 transformed to VGPR, and 1 to PR at the cutoff date. The 9 months PFS rate was 75%. The dose 4.0 × 10^6^ cells/kg was selected for future dose-expansion study. Ten (62.5%) patients experienced grade 1–2 CRS, and 4 patients had reversible grade ≥ 3 CRS. All hematological toxicities were relieved within the first month after infusion. No NTX, DLTs, or deaths were reported [158].

## CAR-NK cells targeting BCMA

Natural killer (NK) cell engineering has recently emerged as a promising alternative approach for cancer therapy. NK cells belong to the innate immune system. Unlike T cells, NK cell activation does not require antigen presentation or strict human leukocyte antigen (HLA) matching. CAR-NK cells do not carry the risk of graft-versus-host disease (GVHD), and they can be generated from NK cell lines (such as NK92) or induced pluripotent stem cells (iPSCs) instead of autologous manufacturing. Therefore, CAR-NK cells can be standardized as an “off-the-shelf” therapy product. In addition, the cytotoxicity of NK cell is mediated via the release of perforin and granzyme as well as the expression of apoptosis-inducing ligands including FasL and TRAIL. So there is much less concern about the CRS seen in many CAR-T cell therapies [159, 160]. Several preclinical studies of CAR-NK cells have been performed in MM. CAR-NK-92MI cells transfected with anti-CD138 scFv presented with enhanced cytotoxicity toward CD138-positive MM cells [161]. CAR-NK 92 cells targeting SLAMF7 not only showed intensified in vitro cytolysis, but also anti-tumor activity in murine tumor models and prolonged mouse survival [162]. A phase 1 trial of a newly developed BCMA-specific CAR-NK 92 cell product has enrolled 20 R/R MM patients aged between 18 and 80 years old in China in May 2019 (NCT03940833). The trial is ongoing with no interim report yet.

## Bispecific T cell engager (BiTE) and trispecific T cell engager (TiTE)

Bispecific T cell engager (BiTE) is a double scFv-containing molecule that binds concomitantly to CD3 and a tumor-specific antigen, facilitating T cell/cancer cell crosslinking, followed by CD4^+^/CD8^+^ T cell activation. The activated T cells secrete interferon-γ, granzyme B, and perforin and exert profound T cell cytotoxicity against tumor cells without requiring antigen-presenting cells, MHC-I/peptide complex, and co-stimulatory molecules [163]. Blinatumomab (BLINCYTO®), the first CD19/CD3 BiTE, has been approved by FDA for the treatment of R/R CD19+ acute lymphocytic leukemia (ALL) [164–166]. BCMA BiTE agents are in clinical trials (Table 4).

**Table 4:** BCMA-targeted bispecific T cell engagers in clinical trials

Name (manufacturer)	Structure	Clinical trial information	Inclusion/exclusion criteria	Pt characteristics	Dosage	Major Response	Most common AE
AMG 420 (Amgen)	BCMA/CD3	Phase 1 NCT02514239 [72]	R/R MM who received or were refractory to ≥ 2 prior lines, including PI and IMiD; PC leukemia, extra-medullary relapse, CNS involvement, or prior ASCT were excluded	42 pts; median age 65; median prior lines 4	0.2–800 μg/d, 4 wks infusion +2 wks off, for up to 5 cycles. Avg 2.5 ± 2.6 cycles	ORR 31%; sCR 14%, CR 7%, VGPR 4.8%, PR 4.8%	G3+ infection 12 (28.5%), polyneuropathy 2 (4.8%); G2–3 CRS 3 (7%); DLT 3 (7%)
CC-93269 (Celgene)	BCMA (bivalent)/CD3 (monovalent)	Phase 1 NCT03486067 [167]	R/R MM who received or were refractory to ≥ 3 prior lines; hx of BCMA-directed therapy were excluded	19 pts; median age 64; median prior lines 6; ASCT 16 (84%); all pts refractory to the last line	0.15–10 mg/d for a 28-day cycle (D1, 8, 15, and 22 for Cycles 1–3; D1 and 15 for Cycles 4–6; and on D1 for Cycle 7). Median 4 cycles; Median DOT 14.6 wks	12 pts w/ dose of ≥ 6 mg; ORR 10 (83.3%); sCR/CR 4 (33.3%), VGPR 7 (58.3%)	G3+ neutropenia (52.6%), anemia (42.1%), infections (26.3%), thrombocytopenia (21.1%); G1–2 CRS 17 (89.5%)
PF-06863135 (Pfizer)	BCMA/CD3, IgG2a backbone	Phase 1 NCT03269136 [168]	R/R MM who received or were refractory to ≥ 3 prior lines, including PI, IMiD, CD38 mAb	17 pts; median age 61; median prior lines 11; 5 pts (29%) had prior BCMA-targeted therapy	Once weekly non-continuous infusion in 6 dose-escalation groups	Minimal response 1 (6%); SD 6 (35%); PD 9 (53%)	G3+ thrombocytopenia (24%), anemia (18%); G1–2 CRS (24%)
REGN5458 (Regeneron)	BCMA/CD3	Phase 1 NCT03761108 [169]	R/R MM who received or were refractory to ≥3 prior lines, including PI, IMiD, CD38 mAb	7 pts	6 mg/kg, 16 weekly doses + maintenance 12 doses per 2 wks	ORR 4 (53.3%)	G1–2 CRS 3 (42.9%)
AMG 701 (Amgen)	BCMA/CD3, extended half-life	Phase 1 NCT03287908	R/R MM who received or were refractory to ≥ 3 prior lines, including PI, IMiD, CD38 mAb	–	–	–	–
TNB383B (TeneoBio)	BCMA (high affinity)/CD3 (low affinity), IgG4 backbone	Phase 1 NCT03933735	R/R MM who received or were refractory to ≥ 3 prior lines, including PI, IMiD, CD38 mAb	–	–	–	–

*Avg* average, *CNS* central nervous system, *CR* complete response, *CRS* cytokine release syndrome, *d* day, *DOT* duration of treatment, *G* grade, *hx* history, *med* median, *ORR* overall response rate, *PC* plasma cells, *PD* progressive disease, *PR* partial response, *pt* patient, *R/R* relapse or refractory, *sCR* stringent complete response, *SD* stable disease, *VGPR* very good partial response, *wk* week

### AMG 420 (BI 836909, Amgen)

AMG 420, formerly BI836909, consists of two linked scFvs, with BCMA scFv positioned N-terminally, and the CD3ε scFv C-terminally followed by a hexahistidine (His6 tag). In vitro study showed that upon simultaneous binding to BCMA+ MM cells and CD3+ T cells, AMG 420 induced crosslinking of both cell types, formation of a cytolytic synapse and subsequently activation of T cells, and release of cytokines (IFNγ, IL-2, IL-6, IL-10, TNFα) in a dose-dependent manner, leading to lysis of BCMA^+^ MM cells. However, BCMA^-^ cells were not affected. The presence of BM stromal cells, sBCMA and APRIL, had very minimal impact on the activity of AMG 420. Ex vivo study showed that AMG 420 induced potent MM cell lysis in both newly diagnosed and R/R MM patient samples. AMG 420 also demonstrated robust tumor-depletion ability in various xenograft models of systemic or subcutaneous MM [170].

A first-in-human phase 1 dose-escalation study (NCT02514239) has started to look at the safety and efficacy of AMG 420 for patients with R/R MM who have failed at least two prior treatment lines including PI and IMiD. Patients with PC leukemia, extra-medullary disease, CNS involvement, or prior ASCT were excluded. In this study, 6-week cycles of AMG 420 (4 weeks continuous IV infusion +2 weeks off) were given for up to 5 cycles or until PD, toxicity, or withdrawal. Five more cycles could be given per investigator for perceived benefit. As of December 10, 2018, 42 patients received AMG 420 (0.2–800 μg/day). Median age was 65, median number of prior therapies was 4, and median number of treatment cycles was 2.5. Overall, 13/42 patients showed responses to treatment (6 sCRs, 3 CRs, 2 VGPRs, 2 PRs). Median time to any response was 1 month. Four hundred micrograms per day was determined to be a recommended dose for further investigation, since this dose led to 7/10 (70%) responses with 5 sCRs, 1 VGPR, and 1 PR. At the data cutoff, the DOR ranged between 5.6 and 10.4 months. Overall, 24 patients had PD. Three DLTs and 2 deaths from AEs were reported. SAEs occurred in 21 (50%) patients including infections (*n* = 12) and polyneuropathy (*n* = 2). Grade 2–3 CRS was also seen in 3 patients. The study is currently still ongoing [72, 73]. AMG 420 has already been granted fast track status by the FDA, and phase 2 development is expected to start.

### CC-93269 (BCMA-TCB2, EM901)

CC-93269 is an asymmetric 2-arm humanized IgG T cell engager that binds bivalently to BCMA and monovalently to CD3 in a 2+1 format. In a preclinical study, CC-93269 promoted MM cell death and tumor regression in different animal models [171, 172]. A phase 1 dose-escalating trial (NCT03486067) evaluating CC-93269 in patients with R/R MM has recently reported its interim updates [167]. Only patients who had received ≥ 3 prior regimens but without prior BCMA-directed therapy were eligible for the study. Intravenous CC-93269 was administered on days 1, 8, 15, and 22 for Cycles 1–3, days 1 and 15 for Cycles 4–6, and day 1 for Cycle 7 and beyond, all in 28-day cycles. As of May 24, 2019, 19 patients with a median age of 64 and a median number of 6 prior treatments received CC-93269 therapy (dose range 0.15–10 mg). Median duration of treatment was 14.6 weeks or 4 cycles. Of the 12 patients treated with a dose of ≥ 6 mg, 10 patients showed responses (ORR 83.3%), including 7 VGPR (58.3%) and 4 sCR/CR (33.3%); 9 (75.0%) patients achieved MRD negativity. The median time to response was 4.2 weeks. One patient presented with PD. Most frequently encountered SAEs were neutropenia (52.6%), anemia (42.1%), infections (26.3%), and thrombocytopenia (21.1%), but no dose modifications were required. CRS were found in 17 (89.5%) patients but were mostly grades 1 and 2. This study is ongoing [167].

### PF-06863135 (PF-3135)

PF-06863135 (PF-3135) is a humanized mAb bispecific to BCMA- and CD3- antigens. PF-3135 exhibited strong cytotoxicity to MM cell lines and different MM primary patient samples. A single injection of PF-3135 effectively inhibited tumor growth in a dose-dependent manner in various established mouse models [173]. A recent update on the ongoing, multicenter, phase 1 trial (NCT03269136) reported 17 patients with R/R MM who were treated with once weekly, non-continuous, IV infusion of PF-3135 in 6 dose-escalation groups. Median age was 61 years old (range 47–82). Median number of prior therapies was 11. Of note, 5 (29%) patients had received prior BCMA-targeted therapy including other BiTEs or CAR-T cells. Among the 16 evaluable patients, 1 (6%) patient had a minimal response and 6 (35%) patients had SD across all dose levels, while 9 (53%) patients had PD. Additional dose cohorts are being enrolled. There were mainly grade 1–2 AEs including CRS (24%), thrombocytopenia (24%), and anemia (18%). DLT of treatment-related febrile neutropenia was found in one patient on the highest dose level [168, 174].

### REGN5458

REGN5458 is another human bispecific antibody against both BCMA and CD3. This agent showed strong cytotoxicity against MM cell lines and primary patient blasts regardless of tumor-surface level of BCMA. REGN5458 was even found to increase the surface level of BCMA. Animal studies also discovered rapid tumor clearance and growth suppression after REGN5458 was administered at doses as low as 0.4 mg/kg. Of note, the anti-tumor activity of REGN5458 was comparable to that of anti-BCMA CAR-T cells both in vitro and in vivo [175]. A phase 1 dose-escalating trial (NCT03761108) is currently investigating the safety and efficacy of REGN5458 in R/R MM. Eligible patients were those who had at least 3 prior regimens of treatment. Sixteen weekly doses of REGN5458 were given first, followed by a maintenance phase of 12 doses administered every 2 weeks. Patients with PD after initial response were allowed to be retreated. According to the most recent update in ASH 2019, 7 patients have received the therapy with 4 responses (ORR 53.3%). Three patients were treated at the dose of 6 mg/kg. In addition, 2 patients achieved MRD-negative status. Regarding safety, no DLTs were reported. Three patients had mild CRS [169].

### AMG 701

AMG 701 is a BiTE similar to AMG 420 but with extended half-life. Preclinical studies showed that AMG 701 markedly induced T cell-mediated lysis of BCMA^+^ MM cells either resistant or sensitive to current anti-MM agents such as IMid or PIs, regardless of the BCMA expression level. It can also trigger robust immunomodulatory effects to overcome the immunocompromised BM microenvironment, such as upregulation of immune checkpoint molecules (PD1, TIM-3, LAG-3), enhanced production of cytokines (INFγ, TNFα), and increased CD8+/CD4+ T cell ratio [176]. AMG 701 in combination with IMiD significantly increased long-term durable responses and reduced relapse in xenograft models [177]. Therefore, AMG 701 alone or in combination with IMiD might improve outcome in MM patients. A phase 1 trial (NCT03287908) is currently ongoing in patients with R/R MM.

### TNB383B

TNB383B is a novel bispecific monoclonal IgG4 antibody that consists of 2 heavy and 1 light chain(s) paired through knob-in-hole technology. The first heavy chain is comprised of two identical scFvs in sequence targeting human BCMA with high affinity and avidity. The second heavy chain and a kappa light chain recognize human CD3 with a low-activating potency. TNB383B was found to have strong ex vivo anti-MM efficacy with markedly reduced cytokine release in preclinical studies [178]. An open-label, multicenter phase 1 trial (NCT03933735) is currently ongoing to evaluate the safety, pharmacokinetics, and efficacy of TNB383B in R/R MM who received at least 3 prior regimens of treatments. No interim result has been reported so far [179].

## BCMA-targeted therapeutics and future perspectives

Even though BCMA-targeted agents achieved remarkable responses in patients with R/R MM, most of them are still at early stage of clinical development. CAR-T therapy has been reported to be effective for extra-medullary plasma cell dyscrasia [71, 115, 130]. However, autologous BCMA CAR-T cells take at least 2–4 weeks to prepare and are mainly available in specialized medical centers, limiting its utility in individuals with rapid PD. For elderly patients (≥ 75 years old) and patients who have been heavily pretreated, it could be difficult to generate sufficient amount of autologous BCMA CAR-T cells [54, 180]. The high incidence of CRS and NTX as well as grade ≥ 3 hematologic AEs associated with the pre-conditioning lymphodepletion are also major concerns, even though the BCMA CAR-T cells were effective for R/R MM patients [124]. A phase 1/2 study (NCT03455972) is currently investigating combined infusion of BCMA- and CD19-specific CAR-T cells in patients with high-risk MM 14 to 20 days after ASCT. Structural modifications have been attempted to decrease the toxicity of CAR-T cell therapy, such as by adding an inhibitory CAR to reduce off-target responses [102, 181], applying a small molecule-gated system to facilitate remote control of CAR-T cells [182], or by attaching suicide genes to the engineered CAR [183]. Several strategies are also proposed to improve efficacy, such as bispecifically targeting both BCMA and another tumor-specific antigen to prevent antigen relapse as discussed above.

BiTE highly relies on the reserved function of T effector cells to generate cytotoxicity. Therefore, it is mainly recommended for patients who have received fewer prior lines of therapy [164, 165]. Compared to CAR-T cells, BiTE has a much shorter half-life; thus, continuous infusion is generally needed [184]. In addition, BiTE also causes SAEs including CRS and NTX. New BiTEs with structural modifications such as AMG 701 and TNB383B have been developed to extend half-life and reduce toxicity. Besides, BiTE combined with IMiD might improve the efficacy as discussed above.

ADC is an “off-the-shelf” product that facilitates direct killing of tumor cells via cytotoxic payloads while carrying a low risk of CRS and NTX. Compared with CAR-T cell product, it is also less expensive and requires less time to prepare. It is given as a bolus infusion in general, not requiring continuous IV administration. However, ADC is cleared by the malignant cells much faster via receptor-mediated endocytosis, thus requiring more frequent infusions. The concern for low payload penetration and bystander effect also limits its anti-tumor ability [80, 81]. Novel cytotoxic agents as payloads (e.g., α-amanitin, tubulysins, hizoxin, spliceostatins) and ADC structure modifications (e.g., non-IgG scaffolds or non-internalizing mAb scaffolds) are currently under development to improve the efficacy [185].

It remains undetermined whether any BCMA-targeted agents (ADC, CAR-T, or BiTE) offers a better therapeutic index. It is equally intriguing whether these agents can be combined concurrently or sequentially. One possible future perspective is that in patients with high MM burden, BCMA ADC could be used as a bridging therapy to quickly reduce tumor load. CAR-T and BiTE could then be administered to eliminate MRD for durable responses. Another question that needs to be addressed in the future is how to appropriately integrate these drugs into current treatment algorithms for MM. Several ongoing clinical trials are currently comparing different combination regimens incorporating BCMA-targeted agents with PIs, IMiDs, checkpoint blockades, or epigenetic inhibitors. The first BCMA-targeted ADC, belantamab mafodotin-blmf (GSK2857916), is already approved for clinical applications for R/R MM. BiTE and CAR-T cells targeting BCMA are promising. The BCMA-targeted immunotherapy is undoubtedly enriching the armamentarium against MM.

## Conclusion

BCMA represents a specific biomarker of normal and malignant PCs. A variety of BCMA-targeted agents including ADC, CAR-T cells, and BiTE are currently in active clinical development. Belantamab mafodotin-blmf (GSK2857916), a BCMA-targeted ADC, has just been approved for highly refractory MM.

## Data Availability

The material supporting the conclusion of this review has been included within the article.

## Abbreviations

ADC: Antibody-drug conjugate
ADCC: Antibody-dependent cell-mediated cytotoxicity
APRIL: A proliferation-inducing ligand
ASCT: Autologous or allogenic stem cell transplantation
BAFF: B cell activating factor
BCMA: B cell maturation antigen
BiTE: Bispecific T cell engager
CAR: Chimeric antigen receptor
CR: Complete response
CRS: Cytokine release syndrome
CTX: Cyclophosphamide
DLT: Dose-limiting toxicity
DOF: Duration of follow-up
FAMP: Fludarabine
IMiD: Immunomodulatory imide drug
mAb: Monoclonal antibodies
mDOR: Median duration of response
MM: Multiple myeloma
mPFS: Median progression-free survival
MRD: Minimal residual disease
MTD: Maximal tolerated dose
NTX: Neurotoxicity
ORR: Overall response rate
OS: Overall survival
PC: Plasma cell
PD: Progressive disease
PI: Proteasome inhibitor
PR: Partial response
R/R: Relapsed and/or refractory
SAE: Severe adverse event
sCR: Stringent complete response
TLS: Tumor lysis syndrome
TRD: Treatment-related death
VGPR: Very good partial response

## References

[1] Raza S, Safyan RA, Lentzsch S (2017). Immunomodulatory drugs (IMiDs) in multiple myeloma. Curr Cancer Drug Targets.

[2] Chehab S, Panjic EH, Gleason C, Lonial S, Nooka AK (2018). Daratumumab and its use in the treatment of relapsed and/or refractory multiple myeloma. Future Oncol.

[3] Le Ray E, Jagannath S, Palumbo A (2016). Advances in targeted therapy for the treatment of patients with relapsed/refractory multiple myeloma. Expert Rev Hematol.

[4] Bray F, Ferlay J, Soerjomataram I, Siegel RL, Torre LA, Jemal A (2018). Global cancer statistics 2018: GLOBOCAN estimates of incidence and mortality worldwide for 36 cancers in 185 countries. CA Cancer J Clin.

[5] Liu J, Liu W, Mi L, Zeng X, Cai C, Ma J, Wang L (2019). Union for China Lymphoma Investigators of the Chinese Society of Clinical O, Union for China Leukemia Investigators of the Chinese Society of Clinical O: Incidence and mortality of multiple myeloma in China, 2006–2016: an analysis of the Global Burden of Disease Study 2016. J Hematol Oncol.

[6] Liu W, Liu J, Song Y, Wang X, Zhou M, Wang L, Ma J, Zhu J (2019). Union for China Leukemia Investigators of the Chinese Society of Clinical Oncology UfCLIotCSoCO: mortality of lymphoma and myeloma in China, 2004–2017: an observational study. J Hematol Oncol.

[7] Lokhorst HM, Plesner T, Laubach JP, Nahi H, Gimsing P, Hansson M, Minnema MC, Lassen U, Krejcik J, Palumbo A, van de Donk NW, Ahmadi T, Khan I, Uhlar CM, Wang J, Sasser AK, Losic N, Lisby S, Basse L, Brun N, Richardson PG (2015). Targeting CD38 with daratumumab monotherapy in multiple myeloma. N Engl J Med.

[8] Palumbo A, Anderson K (2011). Multiple myeloma. N Engl J Med.

[9] Usmani S, Ahmadi T, Ng Y, Lam A, Desai A, Potluri R, Mehra M (2016). Analysis of real-world data on overall survival in multiple myeloma patients with >/=3 prior lines of therapy including a proteasome inhibitor (PI) and an immunomodulatory drug (IMiD), or double refractory to a PI and an IMiD. Oncologist.

[10] Bazarbachi AH, Al Hamed R, Malard F, Harousseau JL, Mohty M (2019). Relapsed refractory multiple myeloma: a comprehensive overview. Leukemia.

[11] Liu D (2019). Cancer biomarkers for targeted therapy. Biomarker Research.

[12] Liu D (2019). CAR-T “the living drugs”, immune checkpoint inhibitors, and precision medicine: a new era of cancer therapy. J Hematol Oncol.

[13] Wu C, Zhang L, Brockman QR, Zhan F, Chen L (2019). Chimeric antigen receptor T cell therapies for multiple myeloma. J Hematol Oncol.

[14] Lin Q, Zhao J, Song Y, Liu D (2019). Recent updates on CAR T clinical trials for multiple myeloma. Mol Cancer.

[15] Avigan D, Rosenblatt J (2014). Current treatment for multiple myeloma. N Engl J Med.

[16] Lee L, Bounds D, Paterson J, Herledan G, Sully K, Seestaller-Wehr LM, Fieles WE, Tunstead J, McCahon L, Germaschewski FM, Mayes PA, Craigen JL, Rodriguez-Justo M, Yong KL (2016). Evaluation of B cell maturation antigen as a target for antibody drug conjugate mediated cytotoxicity in multiple myeloma. Br J Haematol.

[17] Wei J, Han X, Bo J, Han W (2019). Target selection for CAR-T therapy. J Hematol Oncol.

[18] Kozlow EJ, Wilson GL, Fox CH, Kehrl JH (1993). Subtractive cDNA cloning of a novel member of the Ig gene superfamily expressed at high levels in activated B lymphocytes. Blood.

[19] Laabi Y, Gras MP, Brouet JC, Berger R, Larsen CJ, Tsapis A (1994). The BCMA gene, preferentially expressed during B lymphoid maturation, is bidirectionally transcribed. Nucleic Acids Res.

[20] Laabi Y, Gras MP, Carbonnel F, Brouet JC, Berger R, Larsen CJ, Tsapis A (1992). A new gene, BCM, on chromosome 16 is fused to the interleukin 2 gene by a t(4;16)(q26;p13) translocation in a malignant T cell lymphoma. EMBO J.

[21] Zhou LJ, Schwarting R, Smith HM, Tedder TF (1992). A novel cell-surface molecule expressed by human interdigitating reticulum cells, Langerhans cells, and activated lymphocytes is a new member of the Ig superfamily. J Immunol.

[22] Madry C, Laabi Y, Callebaut I, Roussel J, Hatzoglou A, Le Coniat M, Mornon JP, Berger R, Tsapis A (1998). The characterization of murine BCMA gene defines it as a new member of the tumor necrosis factor receptor superfamily. Int Immunol.

[23] Smirnova AS, Andrade-Oliveira V, Gerbase-DeLima M (2008). Identification of new splice variants of the genes BAFF and BCMA. Mol Immunol.

[24] Coquery CM, Erickson LD (2012). Regulatory roles of the tumor necrosis factor receptor BCMA. Crit Rev Immunol.

[25] Marsters SA, Yan M, Pitti RM, Haas PE, Dixit VM, Ashkenazi A (2000). Interaction of the TNF homologues BLyS and APRIL with the TNF receptor homologues BCMA and TACI. Curr Biol.

[26] Gross JA, Johnston J, Mudri S, Enselman R, Dillon SR, Madden K, Xu W, Parrish-Novak J, Foster D, Lofton-Day C, Moore M, Littau A, Grossman A, Haugen H, Foley K, Blumberg H, Harrison K, Kindsvogel W, Clegg CH (2000). TACI and BCMA are receptors for a TNF homologue implicated in B-cell autoimmune disease. Nature.

[27] Thompson JS, Schneider P, Kalled SL, Wang L, Lefevre EA, Cachero TG, MacKay F, Bixler SA, Zafari M, Liu ZY, Woodcock SA, Qian F, Batten M, Madry C, Richard Y, Benjamin CD, Browning JL, Tsapis A, Tschopp J, Ambrose C (2000). BAFF binds to the tumor necrosis factor receptor-like molecule B cell maturation antigen and is important for maintaining the peripheral B cell population. J Exp Med.

[28] Sasaki Y, Casola S, Kutok JL, Rajewsky K, Schmidt-Supprian M (2004). TNF family member B cell-activating factor (BAFF) receptor-dependent and -independent roles for BAFF in B cell physiology. J Immunol.

[29] Seshasayee D, Valdez P, Yan M, Dixit VM, Tumas D, Grewal IS (2003). Loss of TACI causes fatal lymphoproliferation and autoimmunity, establishing TACI as an inhibitory BLyS receptor. Immunity.

[30] Darce JR, Arendt BK, Chang SK, Jelinek DF (2007). Divergent effects of BAFF on human memory B cell differentiation into Ig-secreting cells. J Immunol.

[31] Avery DT, Kalled SL, Ellyard JI, Ambrose C, Bixler SA, Thien M, Brink R, Mackay F, Hodgkin PD, Tangye SG (2003). BAFF selectively enhances the survival of plasmablasts generated from human memory B cells. J Clin Invest.

[32] O'Connor BP, Raman VS, Erickson LD, Cook WJ, Weaver LK, Ahonen C, Lin LL, Mantchev GT, Bram RJ, Noelle RJ (2004). BCMA is essential for the survival of long-lived bone marrow plasma cells. J Exp Med.

[33] Tai YT, Mayes PA, Acharya C, Zhong MY, Cea M, Cagnetta A, Craigen J, Yates J, Gliddon L, Fieles W, Hoang B, Tunstead J, Christie AL, Kung AL, Richardson P, Munshi NC, Anderson KC (2014). Novel anti-B-cell maturation antigen antibody-drug conjugate (GSK2857916) selectively induces killing of multiple myeloma. Blood.

[34] Carpenter RO, Evbuomwan MO, Pittaluga S, Rose JJ, Raffeld M, Yang S, Gress RE, Hakim FT, Kochenderfer JN (2013). B-cell maturation antigen is a promising target for adoptive T-cell therapy of multiple myeloma. Clin Cancer Res.

[35] Deng S, Yuan T, Cheng X, Jian R, Jiang J (2010). B-lymphocyte-induced maturation protein1 up-regulates the expression of B-cell maturation antigen in mouse plasma cells. Mol Biol Rep.

[36] Xu S, Lam KP (2001). B-cell maturation protein, which binds the tumor necrosis factor family members BAFF and APRIL, is dispensable for humoral immune responses. Mol Cell Biol.

[37] Eckhert E, Hewitt R, Liedtke M (2019). B-cell maturation antigen directed monoclonal antibody therapies for multiple myeloma. Immunotherapy.

[38] Bellucci R, Alyea EP, Chiaretti S, Wu CJ, Zorn E, Weller E, Wu B, Canning C, Schlossman R, Munshi NC, Anderson KC, Ritz J (2005). Graft-versus-tumor response in patients with multiple myeloma is associated with antibody response to BCMA, a plasma-cell membrane receptor. Blood.

[39] Claudio JO, Masih-Khan E, Tang H, Goncalves J, Voralia M, Li ZH, Nadeem V, Cukerman E, Francisco-Pabalan O, Liew CC, Woodgett JR, Stewart AK (2002). A molecular compendium of genes expressed in multiple myeloma. Blood.

[40] Tai YT, Acharya C, An G, Moschetta M, Zhong MY, Feng X, Cea M, Cagnetta A, Wen K, van Eenennaam H, van Elsas A, Qiu L, Richardson P, Munshi N, Anderson KC (2016). APRIL and BCMA promote human multiple myeloma growth and immunosuppression in the bone marrow microenvironment. Blood.

[41] Darce JR, Arendt BK, Wu X, Jelinek DF (2007). Regulated expression of BAFF-binding receptors during human B cell differentiation. J Immunol.

[42] Moreaux J, Legouffe E, Jourdan E, Quittet P, Reme T, Lugagne C, Moine P, Rossi JF, Klein B, Tarte K (2004). BAFF and APRIL protect myeloma cells from apoptosis induced by interleukin 6 deprivation and dexamethasone. Blood.

[43] Tai YT, Li XF, Breitkreutz I, Song W, Neri P, Catley L, Podar K, Hideshima T, Chauhan D, Raje N, Schlossman R, Richardson P, Munshi NC, Anderson KC (2006). Role of B-cell-activating factor in adhesion and growth of human multiple myeloma cells in the bone marrow microenvironment. Cancer Res.

[44] Mulazzani M, Huber M, Borchard S, Langer S, Angele B, Schuh E, Meinl E, Dreyling M, Birnbaum T, Straube A, Koedel U, von Baumgarten L (2019). APRIL and BAFF: novel biomarkers for central nervous system lymphoma. J Hematol Oncol.

[45] Day ES, Cachero TG, Qian F, Sun Y, Wen D, Pelletier M, Hsu YM, Whitty A (2005). Selectivity of BAFF/BLyS and APRIL for binding to the TNF family receptors BAFFR/BR3 and BCMA. Biochemistry.

[46] Moreaux J, Sprynski AC, Dillon SR, Mahtouk K, Jourdan M, Ythier A, Moine P, Robert N, Jourdan E, Rossi JF, Klein B (2009). APRIL and TACI interact with syndecan-1 on the surface of multiple myeloma cells to form an essential survival loop. Eur J Haematol.

[47] Bolkun L, Lemancewicz D, Jablonska E, Kulczynska A, Bolkun-Skornicka U, Kloczko J, Dzieciol J (2014). BAFF and APRIL as TNF superfamily molecules and angiogenesis parallel progression of human multiple myeloma. Ann Hematol.

[48] Matthes T, McKee T, Dunand-Sauthier I, Manfroi B, Park S, Passweg J, Huard B (2015). Myelopoiesis dysregulation associated to sustained APRIL production in multiple myeloma-infiltrated bone marrow. Leukemia.

[49] Pan J, Sun Y, Zhang N, Li J, Ta F, Wei W, Yu S, Ai L (2017). Characteristics of BAFF and APRIL factor expression in multiple myeloma and clinical significance. Oncol Lett.

[50] Yaccoby S, Pennisi A, Li X, Dillon SR, Zhan F, Barlogie B, Shaughnessy JD (2008). Atacicept (TACI-Ig) inhibits growth of TACI (high) primary myeloma cells in SCID-hu mice and in coculture with osteoclasts. Leukemia.

[51] Lin L, Cho SF, Wen K, Yu T, Hsieh PA, Li Y, An G, Qiu L, Munshi N, Anderson KC, Tai YT: Impacts of a proliferation-inducing ligand on current therapeutic monoclonal antibody-induced cytotoxicity against human multiple myeloma cells. Blood 2019, 134(Supplement_1):3105.

[52] Demchenko YN, Glebov OK, Zingone A, Keats JJ, Bergsagel PL, Kuehl WM (2010). Classical and/or alternative NF-kappaB pathway activation in multiple myeloma. Blood.

[53] Hua H, Kong Q, Zhang H, Wang J, Luo T, Jiang Y (2019). Targeting mTOR for cancer therapy. J Hematol Oncol.

[54] Tai YT, Anderson KC (2019). B cell maturation antigen (BCMA)-based immunotherapy for multiple myeloma. Expert Opin Biol Ther.

[55] Hatzoglou A, Roussel J, Bourgeade MF, Rogier E, Madry C, Inoue J, Devergne O, Tsapis A (2000). TNF receptor family member BCMA (B cell maturation) associates with TNF receptor-associated factor (TRAF) 1, TRAF2, and TRAF3 and activates NF-kappa B, elk-1, c-Jun N-terminal kinase, and p38 mitogen-activated protein kinase. J Immunol.

[56] Hendriks J, Planelles L, de Jong-Odding J, Hardenberg G, Pals ST, Hahne M, Spaargaren M, Medema JP (2005). Heparan sulfate proteoglycan binding promotes APRIL-induced tumor cell proliferation. Cell Death Differ.

[57] Cassinelli G, Ronchetti D, Laccabue D, Mattioli M, Cuccuru G, Favini E, Nicolini V, Greco A, Neri A, Zunino F, Lanzi C (2009). Concomitant downregulation of proliferation/survival pathways dependent on FGF-R3, JAK2 and BCMA in human multiple myeloma cells by multi-kinase targeting. Biochem Pharmacol.

[58] Shaffer AL, Emre NC, Lamy L, Ngo VN, Wright G, Xiao W, Powell J, Dave S, Yu X, Zhao H, Zeng Y, Chen B, Epstein J, Staudt LM (2008). IRF4 addiction in multiple myeloma. Nature.

[59] Laurent SA, Hoffmann FS, Kuhn PH, Cheng Q, Chu Y, Schmidt-Supprian M, Hauck SM, Schuh E, Krumbholz M, Rubsamen H, Wanngren J, Khademi M, Olsson T, Alexander T, Hiepe F, Pfister HW, Weber F, Jenne D, Wekerle H, Hohlfeld R, Lichtenthaler SF (2015). Meinl E: gamma-Secretase directly sheds the survival receptor BCMA from plasma cells. Nat Commun.

[60] Gutierrez MJ, Desiderio SV, Wang NY, Darrah E, Cappelli L, Nino G, Jones M, Bingham CO (2019). Soluble markers of antibody secreting cell function as predictors of infection risk in rheumatoid arthritis. J Immunol Res.

[61] Sanchez E, Li M, Kitto A, Li J, Wang CS, Kirk DT, Yellin O, Nichols CM, Dreyer MP, Ahles CP, Robinson A, Madden E, Waterman GN, Swift RA, Bonavida B, Boccia R, Vescio RA, Crowley J, Chen H, Berenson JR (2012). Serum B-cell maturation antigen is elevated in multiple myeloma and correlates with disease status and survival. Br J Haematol.

[62] Ghermezi M, Li M, Vardanyan S, Harutyunyan NM, Gottlieb J, Berenson A, Spektor TM, Andreu-Vieyra C, Petraki S, Sanchez E, Udd K, Wang CS, Swift RA, Chen H, Berenson JR (2017). Serum B-cell maturation antigen: a novel biomarker to predict outcomes for multiple myeloma patients. Haematologica.

[63] Ali SA, Shi V, Maric I, Wang M, Stroncek DF, Rose JJ, Brudno JN, Stetler-Stevenson M, Feldman SA, Hansen BG, Fellowes VS, Hakim FT, Gress RE, Kochenderfer JN (2016). T cells expressing an anti–B-cell maturation antigen chimeric antigen receptor cause remissions of multiple myeloma. Blood.

[64] Dettman EJ, Rigat F, Albert J, Barnard R, Birchler M, Deghenhardt Y, DeWall S, Gaye B, He Z, Liu V, Opalinska J: Expression of myeloma cell and soluble B-cell maturation antigen (BCMA) in relapsed and refractory multiple myeloma patients treated with GSK2857916 in BMA117159. Blood 2018, 132(Supplement 1):1977-1977.

[65] Sanchez E, Gillespie A, Tang G, Ferros M, Harutyunyan NM, Vardanyan S, Gottlieb J, Li M, Wang CS, Chen H, Berenson JR (2016). Soluble B-cell maturation antigen mediates tumor-induced immune deficiency in multiple myeloma. Clin Cancer Res.

[66] Chen H, Li M, Xu N, Ng N, Sanchez E, Soof CM, Patil S, Udd K, Bujarski S, Cao J, Hekmati T, Ghermezi M, Zhou M, Wang EY, Tanenbaum EJ, Zahab B, Schlossberg R, Yashar MA, Wang CS, Tang GY, Spektor TM, Berenson JR (2019). Serum B-cell maturation antigen (BCMA) reduces binding of anti-BCMA antibody to multiple myeloma cells. Leuk Res.

[67] Pont MJ, Hill T, Cole GO, Abbott JJ, Kelliher J, Salter AI, Hudecek M, Comstock ML, Rajan A, Patel BKR, Voutsinas JM, Wu Q, Liu L, Cowan AJ, Wood BL, Green DJ (2019). Riddell SR: gamma-secretase inhibition increases efficacy of BCMA-specific chimeric antigen receptor T cells in multiple myeloma. Blood.

[68] Ryan MC, Hering M, Peckham D, McDonagh CF, Brown L, Kim KM, Meyer DL, Zabinski RF, Grewal IS, Carter PJ (2007). Antibody targeting of B-cell maturation antigen on malignant plasma cells. Mol Cancer Ther.

[69] Liu D, Zhao J, Song Y, Luo X, Yang T (2019). Clinical trial update on bispecific antibodies, antibody-drug conjugates, and antibody-containing regimens for acute lymphoblastic leukemia. J Hematol Oncol.

[70] Lonial S, Lee HC, Badros A, Trudel S, Nooka AK, Chari A, Abdallah AO, Callander N, Lendvai N, Sborov D, Suvannasankha A, Weisel K, Karlin L, Libby E, Arnulf B, Facon T, Hulin C, Kortum KM, Rodriguez-Otero P, Usmani SZ, Hari P, Baz R, Quach H, Moreau P, Voorhees PM, Gupta I, Hoos A, Zhi E, Baron J, Piontek T (2020). Belantamab mafodotin for relapsed or refractory multiple myeloma (DREAMM-2): a two-arm, randomised, open-label, phase 2 study. Lancet Oncol.

[71] Raje N, Berdeja J, Lin Y, Siegel D, Jagannath S, Madduri D, Liedtke M, Rosenblatt J, Maus MV, Turka A, Lam LP, Morgan RA, Friedman K, Massaro M, Wang J, Russotti G, Yang Z, Campbell T, Hege K, Petrocca F, Quigley MT, Munshi N, Kochenderfer JN (2019). Anti-BCMA CAR T-cell therapy bb2121 in relapsed or refractory multiple myeloma. N Engl J Med.

[72] Topp M, Duell J, Zugmaier G, Attal M, Moreau P, Langer C, Korenke J, Facon T, Salnikov A, Lesley R, Beutner K, Kalabus J, Rasmussen E, Riemann K, Minella A, Munzert GM, Einsele H: Evaluation of AMG 420, an anti-BCMA bispecific T-cell engager (BiTE) immunotherapy, in R/R multiple myeloma (MM) patients: Updated results of a first-in-human (FIH) phase I dose escalation study. J Clin Oncol 2019, 37(no. 15_suppl ):8007-8007.

[73] Topp M, Duell J, Zugmaier G, Attal M, Moreau P, Langer C, Kroenke J, Facon T, Einsele H, Munzert G: Treatment with AMG 420, an anti-B-cell maturation antigen (BCMA) Bispecific T-Cell Engager (BiTE®) antibody construct, induces minimal residual disease (MRD) negative complete responses in relapsed and/or refractory (R/R) multiple myeloma (MM) patients: results of a first-in-human (FIH) phase i dose escalation study. Blood 2018, 132(Supplement 1):1010.

[74] Trudel S, Lendvai N, Popat R, Voorhees PM, Reeves B, Libby EN, Richardson PG, Anderson LD, Sutherland HJ, Yong K, Hoos A, Gorczyca MM, Lahiri S, He Z, Austin DJ, Opalinska JB, Cohen AD (2018). Targeting B-cell maturation antigen with GSK2857916 antibody-drug conjugate in relapsed or refractory multiple myeloma (BMA117159): a dose escalation and expansion phase 1 trial. Lancet Oncol.

[75] Zhao WH, Liu J, Wang BY, Chen YX, Cao XM, Yang Y, Zhang YL, Wang FX, Zhang PY, Lei B, Gu LF, Wang JL, Yang N, Zhang R, Zhang H, Shen Y, Bai J, Xu Y, Wang XG, ZHang RL, Wei LL, Li ZF, Li ZZ, Geng Y, He Q, ZHuang QC, Fan F, He A, Zhang WG: Updated analysis of a phase 1, open-label study of LCAR-B38M, a chimeric antigen receptor T cell therapy directed against B-cell maturation antigen, in patients with relapsed/refractory multiple myeloma. Blood 2018, 132(Supplement 1):955. 10.1186/s13045-018-0681-6 PMC6302465 30572922

[76] Zhao WH, Liu J, Wang BY, Chen YX, Cao XM, Yang Y, Zhang YL, Wang FX, Zhang PY, Lei B, Gu LF, Wang JL, Yang N, Zhang R, Zhang H, Shen Y, Bai J, Xu Y, Wang XG, Zhang RL, Wei LL, Li ZF, Li ZZ, Geng Y, He Q, Zhuang QC, Fan XH, He AL, Zhang WG (2018). A phase 1, open-label study of LCAR-B38M, a chimeric antigen receptor T cell therapy directed against B cell maturation antigen, in patients with relapsed or refractory multiple myeloma. J Hematol Oncol.

[77] Herrera AF, Molina A (2018). Investigational antibody-drug conjugates for treatment of B-lineage malignancies. Clinical Lymphoma, Myeloma and Leukemia.

[78] Diamantis N, Banerji U (2016). Antibody-drug conjugates--an emerging class of cancer treatment. Br J Cancer.

[79] Aujla A, Aujla R, Liu D (2019). Inotuzumab ozogamicin in clinical development for acute lymphoblastic leukemia and non-Hodgkin lymphoma. Biomarker Research.

[80] Yu B, Liu D (2019). Antibody-drug conjugates in clinical trials for lymphoid malignancies and multiple myeloma. J Hematol Oncol.

[81] Yu B, Liu D (2019). Gemtuzumab ozogamicin and novel antibody-drug conjugates in clinical trials for acute myeloid leukemia. Biomark Res.

[82] Nejadmoghaddam MR, Minai-Tehrani A, Ghahremanzadeh R, Mahmoudi M, Dinarvand R, Zarnani AH (2019). Antibody-drug conjugates: possibilities and challenges. Avicenna J Med Biotechnol.

[83] Kantarjian H, Thomas D, Jorgensen J, Jabbour E, Kebriaei P, Rytting M, York S, Ravandi F, Kwari M, Faderl S, Rios MB, Cortes J, Fayad L, Tarnai R, Wang SA, Champlin R, Advani A, O'Brien S (2012). Inotuzumab ozogamicin, an anti-CD22-calecheamicin conjugate, for refractory and relapsed acute lymphocytic leukaemia: a phase 2 study. Lancet Oncol.

[84] Kantarjian HM, DeAngelo DJ, Stelljes M, Martinelli G, Liedtke M, Stock W, Gokbuget N, O'Brien S, Wang K, Wang T, Paccagnella ML, Sleight B, Vandendries E, Advani AS (2016). Inotuzumab ozogamicin versus standard therapy for acute lymphoblastic leukemia. N Engl J Med.

[85] Trudel S, Lendvai N, Popat R, Voorhees PM, Reeves B, Libby EN, Richardson PG, Hoos A, Gupta I, Bragulat V, He Z, Opalinska JB, Cohen AD (2019). Antibody-drug conjugate, GSK2857916, in relapsed/refractory multiple myeloma: an update on safety and efficacy from dose expansion phase I study. Blood Cancer J.

[86] Lu J, Jiang F, Lu A, Zhang G (2016). Linkers having a crucial role in antibody-drug conjugates. Int J Mol Sci.

[87] Weisel K, Hopkins T, Fecteau D, Bao W, Quigley C, Jewell R, Nichols M, Opalinska J: Dreamm-3: a phase 3, open-label, randomized study to evaluate the efficacy and safety of belantamab mafodotin (GSK2857916) monotherapy compared with pomalidomide plus low-dose dexamethasone (Pom/Dex) in participants with relapsed/refractory multiple myeloma (RRMM). Blood 2019, 134(Supplement_1):1900.

[88] Richardson P, Biswas S, Holkova B, Jackson N, Netherway T, Bao W, Ferron-Brady G, Yeakey A, Shelton C, Ahlers C, Franco S, Ballas M, Paul E, Luptakova K, Gupta I, Opalinska J: Dreamm-5: Platform trial evaluating belantamab mafodotin (a BCMA-directed immuno-conjugate) in combination with novel agents in relapsed or refractory multiple myeloma (RRMM). Blood 2019, 134(Supplement_1):1857.

[89] Pahl A, Lutz C, Hechler T (2018). Amanitins and their development as a payload for antibody-drug conjugates. Drug Discov Today Technol.

[90] Singh RK, Jones RJ, Shirazi F, Hong S, Wang H, Wan J, Kuitase I, Pahl A, Orlowski RZ (2019). HDP-101, a novel bcma-targeted antibody conjugated to α-amanitin, is active against myeloma with preferential efficacy against pre-clinical models of deletion 17p. Clinical Lymphoma, Myeloma and Leukemia.

[91] Pahl A, Ko J, Breunig C, Figueroa V, Lehners N, Baumann A, Palfi A, Mueller C, Lutz C, Hechler T, Kulke M, Goldschmidt H, Raab M: HDP-101: Preclinical evaluation of a novel anti-BCMA antibody drug conjugates in multiple myeloma. J Clin Oncol 2018, 36(no. 15_suppl):e14527.

[92] Kinneer K, Meekin J, Varkey R, Xiao X, Zhong H, Breen S, Hurt E, Thomas S, Flynn M, Hynes P, Bezabeth B, Chen C, Wetzel L, Chen R, Tai YT, Anderson KC, Herbst R, Tice D: Preclinical evaluation of MEDI2228, a BCMA-targeting pyrrolobenzodiazepine-linked antibody drug conjugate for the treatment of multiple myeloma Blood 2017, 130(Supplement 1):3153.

[93] Xing L, Lin L, Yu T, Li Y, Wen K, Cho SF, Hsieh PA, Kinneer K, Munshi N, Anderson KC, Tai YT: Anti-Bcma PBD MEDI2228 combats drug resistance and synergizes with bortezomib and inhibitors to DNA damage response in multiple myeloma. Blood 2019, 134(Supplement_1):1817.

[94] Xing L, Li Y, Lin L, Yu T, Wen K, Cho SF, Hsieh PA, Kinneer K, Munshi N, Anderson KC, Tai YT: MEDI2228, a novel Bcma antibody-PBD conjugate, sensitizes human multiple myeloma cells to NK cell-mediated cytotoxicity and upregulates CD38 expression in MM cells. Blood 2019, 134(Supplement_1):3096.

[95] June CH, Sadelain M (2018). Chimeric antigen receptor therapy. N Engl J Med.

[96] Ma T, Shi J, Liu H (2019). Chimeric antigen receptor T cell targeting B cell maturation antigen immunotherapy is promising for multiple myeloma. Ann Hematol.

[97] Han X, Wang Y, Wei J, Han W (2019). Multi-antigen-targeted chimeric antigen receptor T cells for cancer therapy. J Hematol Oncol.

[98] Liu D, Zhao J, Song Y (2019). Engineering switchable and programmable universal CARs for CAR T therapy. J Hematol Oncol.

[99] Zhao J, Lin Q, Song Y, Liu D (2018). Universal CARs, universal T cells, and universal CAR T cells. J Hematol Oncol.

[100] Zhao J, Song Y, Liu D (2019). Clinical trials of dual-target CAR T cells, donor-derived CAR T cells, and universal CAR T cells for acute lymphoid leukemia. J Hematol Oncol.

[101] Wang J, Hu Y, Huang H (2018). Current development of chimeric antigen receptor T-cell therapy. Stem Cell Investigation.

[102] Wu BX, Song N-J, Riesenberg BP, Li Z (2019). Development of molecular and pharmacological switches for chimeric antigen receptor T cells. Experimental Hematology & Oncology.

[103] Munshi NC, Larry D, Anderson J, Shah N, Jagannath S, Berdeja JG, Lonial S, Raje NS, Siegel DSD, Lin Y, Oriol A, Moreau P, Yakoub-Agha I, Delforge M, Petrocca F, Connarn J, Patel P, Huang L, Campbell TB, Hege K, Miguel JS, Investigators obotKS: Idecabtagene vicleucel (ide-cel; bb2121), a BCMA-targeted CAR T-cell therapy, in patients with relapsed and refractory multiple myeloma (RRMM): Initial KarMMa results. J Clin Oncol 2020, 38(15_suppl):8503-8503.

[104] Shah N, Alsina M, Siegel D, Jagannath S, Madduri D, Kaufman JL, Turka A, Lam LP, Massaro M, Hege K, Petrocca F, Berdeja JG, Raje N: Initial results from a phase 1 clinical study of bb21217, a next-generation anti Bcma CAR T therapy. Blood 2018, 132(Supplement 1):488.

[105] Fan F, Zhao W, Liu J, He A, Chen Y, Cao X, Yang N, Wang B, Zhang P, Zhang Y, Wang F, Lei B, Gu L, Wang X, Zhuang Q, Zhang W: Durable remissions with BCMA-specific chimeric antigen receptor (CAR)-modified T cells in patients with refractory/relapsed multiple myeloma. J Clin Oncol 2017, 35(no. 18_suppl):LBA3001.

[106] Chen L, Xu J, Fu WJ, Jin S, Yang S, Yan S, Wu W, Liu YF, Zhang W, Weng XQ, Wang J, Ding XY, Zhu J, Chen Z, Li J, Hou J, Chen SJ, Mi JQ: Updated phase 1 results of a first-in-human open-label study of Lcar-B38M, a structurally differentiated chimeric antigen receptor T (CAR-T) cell therapy targeting B-cell maturation antigen (Bcma). Blood 2019, 134(Supplement_1):1858.

[107] Berdeja JG, Madduri D, Usmani SZ, Singh I, Zudaire E, Yeh T-M, Allred AJ, Olyslager Y, Banerjee A, Goldberg JD, Schecter J, Geng D, Wu X, Carrasco-Alfonso M, Rizvi S, Fan F, Jakubowiak AJ, Jagannath S: Update of CARTITUDE-1: a phase Ib/II study of JNJ-4528, a B-cell maturation antigen (BCMA)-directed CAR-T-cell therapy, in relapsed/refractory multiple myeloma. J Clin Oncol 2020, 38(15_suppl):8505-8505.

[108] Jie J, Hao S, Jiang S, Li Z, Yang M, Zhang W, Yu K, Xiao J, Meng H, Ma L, He M, Wang W, Huang X, Chen LJ, Xing C, Yuan D, Wang S, Tao R, Dai L, Ma H: Phase 1 trial of the safety and efficacy of fully human anti-Bcma CAR T cells in relapsed/refractory multiple myeloma. Blood 2019, 134(Supplement_1):1863.

[109] Gregory T, Cohen AD, Costello C, Ali SA, Berdeja J, Ostertag E, Martin C, Shedlock D, Resler ML, Spear M, Orlowski R, Patel K: Efficacy and safety of P-Bcma-101 CAR-T cells in patients with relapsed/refractory (r/r) multiple myeloma (MM). Blood 2018, 132(Supplement 1):1012.

[110] Cohen AD, Garfall AL, Stadtmauer EA, Melenhorst JJ, Lacey SF, Lancaster E, Vogl DT, Weiss BM, Dengel K, Nelson A, Plesa G, Chen F, Davis MM, Hwang WT, Young RM, Brogdon JL, Isaacs R, Pruteanu-Malinici I, Siegel DL, Levine BL, June CH, Milone MC (2019). B cell maturation antigen-specific CAR T cells are clinically active in multiple myeloma. J Clin Invest.

[111] Li C, Wang J, Hu G, Yang Y, Zhou X, Meng L, Hong Z, Chen L, Mao X, Xiao M, Zhou J: Efficacy and safety of fully human Bcma targeting CAR T cell therapy in relapsed/refractory multiple myeloma. Blood 2019, 134(Supplement_1):929.

[112] Mailankody S, Htut M, Lee K, Bensinger W, Devries T, Piasecki J, Ziyad S, Blake M, Byon J, Jakubowiak A: JCARH125, Anti-BCMA CAR T-cell therapy for relapsed/refractory multiple myeloma: initial proof of concept results from a phase 1/2 multicenter study (EVOLVE). Blood 2018, 132(Supplement 1):957.

[113] Mailankody S, Ghosh A, Staehr M, Purdon TJ, Roshal M, Halton E, Diamonte C, Pineda J, Anant P, Bernal Y, Wills A, Korde N, Lendvai N, Lesokhin AM, Hassoun H, Hultcrantz M, Landau HJ, Shah GL, Scordo M, Chung DJ, Lahoud OB, Khattar P, Gao Q, Jungbluth A, Park JH, Curran KJ, Sauter CS, Palomba ML, Senechal B, Wang X et al: Clinical responses and pharmacokinetics of MCARH171, a human-derived Bcma targeted CAR T cell therapy in relapsed/refractory multiple myeloma: final results of a phase i clinical trial. Blood 2018, 132(Supplement 1):959.

[114] Fu WJ, Du J, Jiang H, Cheng Z, Wei R, Yu K, Jiang S, He F, Fang H, Liu Y, Wang P: Efficacy and safety of CAR-T therapy with safety switch targeting bcma for patients with relapsed/refractory multiple myeloma in a phase 1 clinical study. Blood 2019, 134(Supplement_1):3154.

[115] Xu J, Wang Q, Xu H, Gu C, Jiang L, Wang J, Wang D, Xu B, Mao X, Wang J, Wang Z, Xiao Y, Zhang Y, Li C, Zhou J (2018). Anti-BCMA CAR-T cells for treatment of plasma cell dyscrasia: case report on POEMS syndrome and multiple myeloma. J Hematol Oncol.

[116] Liu D, Zhao J (2018). Cytokine release syndrome: grading, modeling, and new therapy. J Hematol Oncol.

[117] Porter D, Frey N, Wood PA, Weng Y, Grupp SA (2018). Grading of cytokine release syndrome associated with the CAR T cell therapy tisagenlecleucel. J Hematol Oncol.

[118] Neelapu SS, Tummala S, Kebriaei P, Wierda W, Gutierrez C, Locke FL, Komanduri KV, Lin Y, Jain N, Daver N, Westin J, Gulbis AM, Loghin ME, de Groot JF, Adkins S, Davis SE, Rezvani K, Hwu P, Shpall EJ (2018). Chimeric antigen receptor T-cell therapy - assessment and management of toxicities. Nat Rev Clin Oncol.

[119] Neelapu SS, Tummala S, Kebriaei P, Wierda W, Locke FL, Lin Y, Jain N, Daver N, Gulbis AM, Adkins S, Rezvani K, Hwu P, Shpall EJ (2018). Toxicity management after chimeric antigen receptor T cell therapy: one size does not fit 'ALL'. Nat Rev Clin Oncol.

[120] Wang Z, Han W (2018). Biomarkers of cytokine release syndrome and neurotoxicity related to CAR-T cell therapy. Biomarker Research.

[121] Giavridis T, van der Stegen SJC, Eyquem J, Hamieh M, Piersigilli A, Sadelain M (2018). CAR T cell-induced cytokine release syndrome is mediated by macrophages and abated by IL-1 blockade. Nat Med.

[122] Norelli M, Camisa B, Barbiera G, Falcone L, Purevdorj A, Genua M, Sanvito F, Ponzoni M, Doglioni C, Cristofori P, Traversari C, Bordignon C, Ciceri F, Ostuni R, Bonini C, Casucci M, Bondanza A (2018). Monocyte-derived IL-1 and IL-6 are differentially required for cytokine-release syndrome and neurotoxicity due to CAR T cells. Nat Med.

[123] Gagelmann N, Ayuk FA, Atanackovic D, Kroeger N: B cell maturation antigen-specific CAR T Cells for relapsed or refractory multiple myeloma: a meta-analysis. Blood 2019, 134(Supplement_1):3113. 10.1111/ejh.13380 31883150

[124] Brudno JN, Maric I, Hartman SD, Rose JJ, Wang M, Lam N, Stetler-Stevenson M, Salem D, Yuan C, Pavletic S, Kanakry JA, Ali SA, Mikkilineni L, Feldman SA, Stroncek DF, Hansen BG, Lawrence J, Patel R, Hakim F, Gress RE, Kochenderfer JN (2018). T cells genetically modified to express an anti-B-cell maturation antigen chimeric antigen receptor cause remissions of poor-prognosis relapsed multiple myeloma. J Clin Oncol.

[125] Friedman KM, Garrett TE, Evans JW, Horton HM, Latimer HJ, Seidel SL, Horvath CJ, Morgan RA (2018). Effective targeting of multiple B-cell maturation antigen-expressing hematological malignances by anti-B-cell maturation antigen chimeric antigen receptor T cells. Hum Gene Ther.

[126] Thompson E, Jiang Y, Campbell T, Fuller J, Kaiser S, Mashadi-Hossein A, Rytlewski J, Martin N, Finney O, Kleinsteuber K, Alonzo E, Pandya C, Agarwal A, Hege K, Raje N, Munshi N, Hause R: Markers of initial and long-term responses to idecabtagene vicleucel (Ide-Cel; bb2121) in the CRB-401 Study in Relapsed/Refractory Multiple Myeloma. Blood 2019, 134(Supplement_1):4328.

[127] Berdeja JG, Lin Y, Raje NS, Siegel DSD, Munshi NC, Liedtke M, Jagannath S, Maus MV, Turka A, Lam LP, Hege K, Morgan R, Quigley MT, Kochenderfer J: First-in-human multicenter study of bb2121 anti-BCMA CAR T-cell therapy for relapsed/refractory multiple myeloma: Updated results. J Clin Oncol 2017, 35(15_suppl):3010-3010.

[128] Berdeja J, Alsina M, Shah N, Siegel D, Jagannath S, Madduri D, Kaufman JL, Munshi N, Rosenblatt J, Jasielec JK, Lin Y, Turka A, Lam LP, Massaro M, Campbell TB, Hege K, Petrocca F, Raje NS: Updated results from an ongoing phase 1 clinical study of bb21217 Anti-Bcma CAR T cell therapy. Blood 2019, 134(Supplement_1):927.

[129] Wang B, Zhao W, Liu J, Chen Y, Cao X, Yang Y, Zhang Y, Wang F, Zhang P, Lei B, Gu L, Wang J, Zhang J, Zhang R, Zhang H, Shen Y, Bai J, Xu Y, Wang X, Zhang R, Wei L, Li Z, He G, Geng Y, He Q, Zhuang Q, Fan F, Zhang W, He A: Long-term follow-up of a phase 1, first-in-human open-label study of LCAR-B38M, a structurally differentiated chimeric antigen receptor T (CAR-T) cell therapy targeting B-cell maturation antigen (BCMA), in patients (pts) with relapsed/refractory multiple myeloma (RRMM). Blood 2019, 134(Supplement_1):579.

[130] Xu J, Chen LJ, Yang SS, Sun Y, Wu W, Liu YF, Xu J, Zhuang Y, Zhang W, Weng XQ, Wu J, Wang Y, Wang J, Yan H, Xu WB, Jiang H, Du J, Ding XY, Li B, Li JM, Fu WJ, Zhu J, Zhu L, Chen Z, Fan XF, Hou J, Li JY, Mi JQ, Chen SJ (2019). Exploratory trial of a biepitopic CAR T-targeting B cell maturation antigen in relapsed/refractory multiple myeloma. Proc Natl Acad Sci U S A.

[131] Madduri D, Usmani SZ, Jagannath S, Singh I, Zudaire E, Yeh TM, Allred AJ, Banerjee A, Goldberg JD, Scheter JM, Zhuang S, Infante JR, Rizvi S, Fan F, Jakubowiak A, Berdeja J: Results from CARTITUDE-1: a phase 1b/2 study of JNJ-4528, a CAR-T cell therapy directed against B-cell maturation antigen (BCMA), in patients with relapsed and/or refractory multiple myeloma (R/R MM). Blood 2019, 134(Supplement_1):577.

[132] Zudaire E, Madduri D, Usmani S, Jakubowiak A, Berdeja J, Geng D, Rizvi S, Nesheiwat T, Scheter JM, Goldberg JD, Banerjee A, Allred AJ, Singh I, Verona R, McCaffery I, Jagannath S: Translational analysis from CARTITUDE-1, an ongoing phase 1b/2 study of JNJ-4528 BCMA-targeted CAR-T cell therapy in relapsed and/or refractory multiple myeloma (R/R MM), indicates preferential expansion of CD8+ T cell central memory cell subset. Blood 2019, 134(Supplement_1):928.

[133] Jiang S, Jin J, Hao S, Yang M, Chen LJ, Ruan H, Xiao J, Wang W, Li Z, Yu K: Low dose of human scFv-derived BCMA-targeted CAR-T cells achieved fast response and high complete remission in patients with relapsed/refractory multiple myeloma. Blood 2018, 132(Supplement 1):960.

[134] Zhao S, Jiang E, Chen S, Gu Y, Shangguan AJ, Lv T, Luo L, Yu Z (2016). PiggyBac transposon vectors: the tools of the human gene encoding. Translational lung cancer research.

[135] Hermanson D, Barnett BE, Rengarajan S, Codde R, Wang X, Tan Y, Martin C, Smith JB, He J, Mathur R, Yan J, Neelapu S, Osertag E, Shedlock D (2016). A novel Bcma-specific, centyrin-based CAR-T product for the treatment of multiple myeloma. Blood.

[136] Costello C, Gregory T, Ali SA, Berdeja J, Patel K, Shah N, Ostertag E, Martin C, Ghoddusi M, Shedlock D, Spear M, Orlowski R, Cohen AD: Phase 2 study of the response and safety of P-Bcma-101 CAR-T cells in patients with relapsed/refractory (r/r) multiple myeloma (MM) (PRIME). Blood 2019, 134(Supplement_1):3184.

[137] Bu DX, Singh R, Choi EE, Ruella M, Nunez-Cruz S, Mansfield KG, Bennett P, Barton N, Wu Q, Zhang J, Wang Y, Wei L, Cogan S, Ezell T, Joshi S, Latimer KJ, Granda B, Tschantz WR, Young RM, Huet HA, Richardson CJ, Milone MC (2018). Pre-clinical validation of B cell maturation antigen (BCMA) as a target for T cell immunotherapy of multiple myeloma. Oncotarget.

[138] Li C, Zhou J, Wang J, Hu G, Du A, Zhou X, Meng L, Hong Z, Chen L, Mao X: Clinical responses and pharmacokinetics of fully human BCMA targeting CAR T-cell therapy in relapsed/refractory multiple myeloma. J Clin Oncol 2019, 37(no. 15_suppl ):8013-8013.

[139] Harrington K, Wu R, Hauskins C, Amin R, Long T, Chen A, Radardjo A, Thayer C, Navvaro G, Myers M, Jones J, Baturevych A, Morkowski S, Salmon R, Bond C, Staehr M, Purdon TJ, Masakayan R, Liu C, Liu H, Yu Y, Wang P, Pont MJ, Green D, Brentjens RJ, Smith EL, Sather BD: Development of JCARH125: Optimization of a fully human anti-Bcma CAR for use in the treatment of multiple myeloma. Blood 2017, 130(Supplement 1):1813.

[140] Smith EL, Staehr M, Masakayan R, Tatake IJ, Purdon TJ, Wang X, Wang P, Liu H, Xu Y, Garrett-Thomson SC, Almo SC, Riviere I, Liu C, Brentjens RJ (2018). Development and evaluation of an optimal human single-chain variable fragment-derived BCMA-targeted CAR T cell vector. Mol Ther.

[141] Adam G, Feng J, Ghogha A, Mardiros A, Rodriguez R, Spindler T, Wiltzius J, Polverino T: Selectivity and specificity of engineered T cells expressing KITE-585, a chimeric antigen receptor targeting B-cell maturation antigen (BCMA). Cancer Res 2017, 77(13 Supplement):Abstract nr 2135.

[142] Adam G, Feng J, Ghogha A, Mardiros A, Murakami J, Phung T, Rodriguez R, Sievers S, Spindler T, Wiltzius J, Yarka C, Yoder S, Polverino T: Development of KITE-585: a fully human BCMA CAR T-cell therapy for the treatment of multiple myeloma. Cancer Res 2017, 77(13 Supplement):Abstract nr 4979.

[143] Cornell R, Locke FL, Bishop MR, Orlowski R, Larson M, Borrello I, Giralt S, Kumar S, Nooka A, Raje N, Rossi J, Thomrongsith L, Xue A, Roberts Z: A phase 1 multicenter study evaluating KITE-585, an autologous anti-BCMA CAR T-cell therapy, in patients with relapsed/refractory multiple myeloma. J Clin Oncol 2018, 36(no. 15_suppl):TPS3103.

[144] Jia H, Wang Z, Wang Y, Liu Y, Dai H, Tong C, Guo Y, Guo B, Ti D, Han X, Yang Q, Wu Z, Han W (2019). Haploidentical CD19/CD22 bispecific CAR-T cells induced MRD-negative remission in a patient with relapsed and refractory adult B-ALL after haploidentical hematopoietic stem cell transplantation. J Hematol Oncol.

[145] Del Nagro CJ, Otero DC, Anzelon AN, Omori SA, Kolla RV, Rickert RC (2005). CD19 function in central and peripheral B-cell development. Immunol Res.

[146] Wang K, Wei G, Liu D (2012). CD19: a biomarker for B cell development, lymphoma diagnosis and therapy. Experimental Hematology & Oncology.

[147] Garfall AL, Stadtmauer EA, Hwang WT, Lacey SF, Melenhorst JJ, Krevvata M, Carroll MP, Matsui WH, Wang Q, Dhodapkar MV, Dhodapkar K, Das R, Vogl DT, Weiss BM, Cohen AD, Mangan PA, Ayers EC, Nunez-Cruz S, Kulikovskaya I, Davis MM, Lamontagne A, Dengel K, Kerr ND, Young RM, Siegel DL, Levine BL, Milone MC, Maus MV, June CH (2019). Anti-CD19 CAR T cells with high-dose melphalan and autologous stem cell transplantation for refractory multiple myeloma. JCI Insight.

[148] Garfall AL, Stadtmauer EA, Hwang WT, Lacey SF, Melenhorst JJ, Krevvata M, Carroll MP, Matsui WH, Wang Q, Dhodapkar MV, Dhodapkar K, Das R, Vogl DT, Weiss BM, Cohen AD, Mangan PA, Ayers EC, Nunez-Cruz S, Kulikovskaya I, Davis MM, Lamontagne A, Dengel K, Kerr ND, Young RM, Siegel DL, Levine BL, Milone MC, Maus MV, June CH (2018). Anti-CD19 CAR T cells with high-dose melphalan and autologous stem cell transplantation for refractory multiple myeloma. JCI Insight.

[149] Garfall AL, Stadtmauer EA, June CH (2016). Chimeric antigen receptor T cells in myeloma. N Engl J Med.

[150] Garfall AL, Maus MV, Hwang WT, Lacey SF, Mahnke YD, Melenhorst JJ, Zheng Z, Vogl DT, Cohen AD, Weiss BM, Dengel K, Kerr ND, Bagg A, Levine BL, June CH, Stadtmauer EA (2015). Chimeric antigen receptor T cells against CD19 for multiple myeloma. N Engl J Med.

[151] Timmers M, Roex G, Wang Y, Campillo-Davo D, Van Tendeloo VFI, Chu Y, Berneman ZN, Luo F, Van Acker HH, Anguille S (2019). Chimeric antigen receptor-modified T cell therapy in multiple myeloma: beyond B cell maturation antigen. Front Immunol.

[152] Yan Z, Cao J, Cheng H, Qiao J, Zhang H, Wang Y, Shi M, Lan J, Fei X, Jin L, Jing G, Sang W, Zhu F, Chen W, Wu Q, Yao Y, Wang G, Zhao J, Zheng J, Li Z, Xu K (2019). A combination of humanised anti-CD19 and anti-BCMA CAR T cells in patients with relapsed or refractory multiple myeloma: a single-arm, phase 2 trial. Lancet Haematol.

[153] Garfall AL, Cohen AD, Lacey SF, Tian L, Hwang WT, Vogl DT, Waxman A, Lancaster E, Nelson A, Ferthio R, Fesnak A, Lamontagne A, Brennan A, Guilliams J, Glandney WL, Melenhorst JJ, Young RM, Siegel D, Levine BL, Brogdon JL, Isaacs R, June CH, Milone MC, Stadtmauer EA: Combination anti-Bcma and anti-CD19 CAR T cells as consolidation of response to prior therapy in multiple myeloma. Blood 2019, 134(Supplement_1):1863.

[154] Zhang H, Gao L, Liu L, Wang J, Wang S, Gao L, Zhang C, Liu YF, Kong P, Liu J, He J, Han Y, Shi H, He Y, Ye X, Zhao Y, Cao W, Shen L, Zhang X: A Bcma and CD19 bispecific CAR-T for relapsed and refractory multiple myeloma. Blood 2019, 134(Supplement_1):3147.

[155] Morandi F, Horenstein AL, Costa F, Giuliani N, Pistoia V, Malavasi F (2018). CD38: A target for immunotherapeutic approaches in multiple myeloma. Front Immunol.

[156] Ma X, Wong SW, Zhou P, Chaulagain CP, Doshi P, Klein AK, Sprague K, Kugelmass A, Toskic D, Warner M, Miller KB, Lee L, Varga C, Comenzo RL (2018). Daratumumab binds to mobilized CD34+ cells of myeloma patients in vitro without cytotoxicity or impaired progenitor cell growth. Experimental Hematology & Oncology.

[157] Usmani SZ, Khan I, Chiu C, Foureau D, Druhan LJ, Rigby K, Casneuf T, Sasser AK (2018). Deep sustained response to daratumumab monotherapy associated with T-cell expansion in triple refractory myeloma. Experimental Hematology & Oncology.

[158] Li C, Mei H, Hu Y, Guo T, Liu L, Jiang H, Tang L, Wu Y, Ai L, Deng J, Jin D: A bispecific CAR-T cell therapy targeting Bcma and CD38 for relapsed/refractory multiple myeloma: updated results from a phase 1 dose-climbing trial. Blood 2019, 134(Supplement_1):930.

[159] Rezvani K, Rouce R, Liu E, Shpall E (2017). Engineering natural killer cells for cancer immunotherapy. Mol Ther.

[160] Siegler EL, Zhu Y, Wang P, Yang L (2018). Off-the-Shelf CAR-NK cells for cancer immunotherapy. Cell Stem Cell.

[161] Jiang H, Zhang W, Shang P, Zhang H, Fu W, Ye F, Zeng T, Huang H, Zhang X, Sun W, Man-Yuen Sze D, Yi Q, Hou J (2014). Transfection of chimeric anti-CD138 gene enhances natural killer cell activation and killing of multiple myeloma cells. Mol Oncol.

[162] Chu J, Deng Y, Benson DM, He S, Hughes T, Zhang J, Peng Y, Mao H, Yi L, Ghoshal K, He X, Devine SM, Zhang X, Caligiuri MA, Hofmeister CC, Yu J (2014). CS1-specific chimeric antigen receptor (CAR)-engineered natural killer cells enhance in vitro and in vivo antitumor activity against human multiple myeloma. Leukemia.

[163] Holliger P, Prospero T, Winter G (1993). “Diabodies”: small bivalent and bispecific antibody fragments. Proc Natl Acad Sci U S A.

[164] Zhao J, Song Y, Liu D (2019). Recent advances on blinatumomab for acute lymphoblastic leukemia. Experimental Hematology & Oncology.

[165] Kantarjian H, Stein A, Gökbuget N, Fielding AK, Schuh AC, Ribera J-M, Wei A, Dombret H, Foà R, Bassan R, Arslan Ö, Sanz MA, Bergeron J, Demirkan F, Lech-Maranda E, Rambaldi A, Thomas X, Horst H-A, Brüggemann M, Klapper W, Wood BL, Fleishman A, Nagorsen D, Holland C, Zimmerman Z, Topp MS (2017). Blinatumomab versus chemotherapy for advanced acute lymphoblastic leukemia. N Engl J Med.

[166] Newman MJ, Benani DJ (2016). A review of blinatumomab, a novel immunotherapy. J Oncol Pharm Pract.

[167] Costa L, Wong S, Bermudez A, Rubia J, Mateos MV, Ocio E, Rodriguez-Otero P, San-Miguel J, Li S, Sarmiento R, Lardelli P, Gaudy A, Boss I, Kelly L, Burgess M, Hege K, Bensinger W: First clinical study of the B-cell maturation antigen (BCMA) 2+1 T cell engager (TCE) CC-93269 in patients (Pts) with relapsed/refractory multiple myeloma (RRMM): interim results of a phase 1 multicenter trial. Blood 2019, 134(Supplement_1):143.

[168] Raje N, Jakubowiak A, Gasparetto C, Cornell R, Krupka H, Navarro D, Forgie A, Udata C, Basu C, Chou J, Leung A, Lesokhin AM: Safety, clinical activity, pharmacokinetics, and pharmacodynamics from a phase i study of PF-06863135, a B-cell maturation antigen (BCMA)-CD3 bispecific antibody, in patients with relapsed/refractory multiple myeloma (RRMM). Blood 2019, 134(Supplement 1):1869.

[169] Cooper D, Madduri D, Lentzsch S, Jagannath S, Li J, Boyapati A, Adriaens L, Choski D, Lowy I, Weinereich D, Yangcopoulos G, Sharma M, Karasarides M, Sternber D: Safety and preliminary clinical activity of REGN5458, an anti-Bcma x anti-CD3 bispecific antibody, in patients with relapsed/refractory multiple myeloma. Blood 2019, 134(Supplement_1):3178.

[170] Hipp S, Tai YT, Blanset D, Deegen P, Wahl J, Thomas O, Rattel B, Adam PJ, Anderson KC, Friedrich M (2017). A novel BCMA/CD3 bispecific T-cell engager for the treatment of multiple myeloma induces selective lysis in vitro and in vivo. Leukemia.

[171] Seckinger A, Delgado JA, Moser S, Moreno L, Neuber B, Grab A, Lipp S, Merino J, Prosper F, Emde M, Delon C, Latzko M, Gianotti R, Luoend R, Murr R, Hosse RJ, Harnisch LJ, Bacac M, Fauti T, Klein C, Zabaleta A, Hillengass J, Cavalcanti-Adam EA, Ho AD, Hundemer M, San Miguel JF, Strein K, Umana P, Hose D, Paiva B (2017). Target expression, generation, preclinical activity, and pharmacokinetics of the BCMA-T cell bispecific antibody EM801 for multiple myeloma treatment. Cancer Cell.

[172] Hagner PR, Waldman M, Gray FD, Yura R, Hersey S, Chan H, Zhang M, Boss I, Gandhi AK: Targeting B-cell maturation antigen (BCMA) with CC-93269, a 2+1 T cell engager, elicits significant apoptosis in diffuse large B-cell lymphoma preclinical models. Blood 2019, 134(Supplement_1):1580-1580.

[173] Panowski S, Kuo T, Chen A, Geng T, Blarcom T, Lindquist K, Chen W, Chaparro-Riggers J, Sasu B (2016). Preclinical evaluation of a potent anti-Bcma CD3 bispecific molecule for the treatment of multiple myeloma. Blood.

[174] Lesokhin AM, Raje N, Gasparetto C, Walker J, Krupka H, Joh T, Taylor C, Jakubowiak A: A phase i, open-label study to evaluate the safety, pharmacokinetic, pharmacodynamic, and clinical activity of PF-06863135, a B-cell maturation antigen/CD3 bispecific antibody, in patients with relapsed/refractory advanced multiple myeloma. Blood 2018, 132(Supplement 1):3229.

[175] Dilillo D, Olson K, Mohrs K, Meagher C, Bray K, Sineschchekova O, Startz T, Retter M, Godin S, Delfino F, J. L, Smith E, Thurston OG, Kirshner J: REGN5458, a bispecific BCMAxCD3 T cell engaging antibody, demonstrates robust in vitro and in vivo anti-tumor efficacy in multiple myeloma models, comparable to that of BCMA CAR T cells. Blood 2018, 132(Supplement 1):1944.

[176] Cho SF, Lin L, Xing L, Liu J, Yu T, Wen K, Hsieh PA, Munshi N, Anderson KC, Tai YT: Anti-BCMA BiTE® AMG 701 potently induces specific T cell lysis of human multiple myeloma (MM) cells and immunomodulation in the bone marrow microenvironment. Blood 2018, 132(Supplement 1):592.

[177] Cho SF, Lin L, Xing L, Wen K, Yu T, Hsieh PA, Li Y, Munshi N, Wahl J, Matthes T, Anderson KC, Chapman-Arvedson T, Tai YT: AMG 701 potently induces anti-multiple myeloma (MM) functions of T cells and IMiDs further enhance its efficacy to prevent MM relapse in vivo. Blood 2019, 134(Supplement_1):135.

[178] Fourearu D, Bhutani M, Robinson M, Guo F, Pham D, Aldred SF, Buelow B, Rigby K, Tjaden E, Leonidas M, Atrash S, Ndiaye A, Symanowski J, Voorhees PM, Usmani SZ: Ex vivo assessment of Tnb-383B, a Bcma-bispecific antibody, against primary tumor and endogenous T cells from relapsing multiple myeloma patients. Blood 2018, 132(Supplement 1):1940.

[179] Buelow B, D'Souza A, Rodriguez C, Vij R, Nath R, Snyder M, Pham D, Patel A, Iyer S: TNB383B.0001: A multicenter, phase 1, open-label, dose-escalation andexpansion study of TNB-383B, a bispecific antibodytargeting BCMA in subjects with relapsed or refractorymultiple myeloma. Blood 2019, 134(Supplement_1):1874.

[180] Nie Y, Lu W, Chen D, Tu H, Guo Z, Zhou X, Li M, Tu S, Li Y (2020). Mechanisms underlying CD19-positive ALL relapse after anti-CD19 CAR T cell therapy and associated strategies. Biomarker Research.

[181] Fedorov VD, Themeli M, Sadelain M (2013). PD-1- and CTLA-4-based inhibitory chimeric antigen receptors (iCARs) divert off-target immunotherapy responses. Sci Transl Med.

[182] Wu CY, Roybal KT, Puchner EM, Onuffer J, Lim WA (2015). Remote control of therapeutic T cells through a small molecule-gated chimeric receptor. Science.

[183] Quintarelli C, Vera JF, Savoldo B, Giordano Attianese GM, Pule M, Foster AE, Heslop HE, Rooney CM, Brenner MK, Dotti G (2007). Co-expression of cytokine and suicide genes to enhance the activity and safety of tumor-specific cytotoxic T lymphocytes. Blood.

[184] Klinger M, Brandl C, Zugmaier G, Hijazi Y, Bargou RC, Topp MS, Gokbuget N, Neumann S, Goebeler M, Viardot A, Stelljes M, Bruggemann M, Hoelzer D, Degenhard E, Nagorsen D, Baeuerle PA, Wolf A, Kufer P (2012). Immunopharmacologic response of patients with B-lineage acute lymphoblastic leukemia to continuous infusion of T cell-engaging CD19/CD3-bispecific BiTE antibody blinatumomab. Blood.

[185] Cho SF, Anderson KC, Tai YT (2018). Targeting B cell maturation antigen (BCMA) in multiple myeloma: potential uses of BCMA-based immunotherapy. Front Immunol.

